# Discerning Mode-Controlled Relaxation Pathways in Photoionized Methane via X‑ray Transient Absorption Spectroscopy

**DOI:** 10.1021/jacs.6c07197

**Published:** 2026-08-13

**Authors:** Kevin Marin, Francesco A. Evangelista

**Affiliations:** Department of Chemistry and Cherry Emerson Center for Scientific Computation, 1371 Emory University, Atlanta 30322, Georgia, United States

## Abstract

Deciphering ultrafast relaxation pathways following the excitation of specific vibrational modes can inform our understanding of mode-selective chemistry; however, direct spectroscopic signatures of the underlying nuclear dynamics remain elusive. Here, we show that X-ray transient absorption spectroscopy at the carbon K-edge can resolve how vibrational pre-excitation biases the ultrafast dynamics of CH_4_^+^ following strong-field ionization of methane. We utilize *ab initio* molecular dynamics combined with high-level multireference computations of core-excited states to track these femtosecond dynamics starting from methane excited in its asymmetric stretching (ν_3_ = 1) and asymmetric scissoring (ν_4_ = 1) modes. Our simulations show that core excitation to the singly occupied molecular orbital (SOMO) tracks bond angles, whereas core excitation to the lowest unoccupied molecular orbital (LUMO) reports on bond lengths. These spectroscopic signatures distinguish two major mode-selective relaxation pathways: asymmetric stretching excitation promotes the formation of a transient CH_3_^+^···H complex, whereas asymmetric scissoring enhances angular distortion along the Jahn–Teller distortion coordinate, increasing sampling of C_2v_ CH_4_^+^ geometries. These results identify for each relaxation pathway an experimentally testable X-ray signature of vibrationally-steered ionization dynamics in methane.

## Graphical abstract

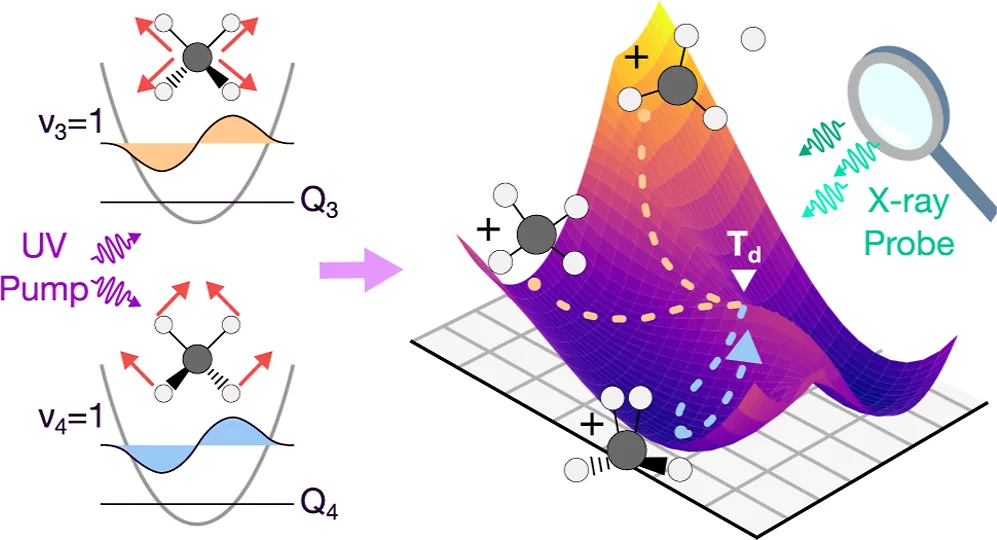

## Introduction

Controlling chemical reactions by exciting specific vibrational modes is one of the foundational ambitions of mode-selective chemistry.^1–5^ Realizing this control requires experimental handles that can track the reaction unfolding in initial stages, with sufficient temporal resolution to correlate excitation modes to the nuclear dynamics in the critical first few tens of femtoseconds. X-ray transient absorption spectroscopy (XTAS) is particularly attractive for resolving nuclear motion in the ultrafast regime due to its element specificity and femtosecond resolution.^6–14^ Although XTAS has been extensively utilized in pump–probe experiments to study photochemical mechanisms,^15–33^ it is yet to be applied to determine how selective vibrational excitation influences subsequent relaxation dynamics and whether those differences can be resolved spectroscopically.

The methane cation is an ideal system for exploring mode-driven control in the context of Jahn–Teller (JT)^34^ symmetry breaking.^35–40^ Using attosecond transient absorption spectroscopy (ATAS), Ridente and co-workers^23^ measured the carbon K-edge of CH_4_^+^ to investigate the symmetry-breaking JT dynamics following strong-field ionization (SFI) of methane. Their combined experimental and computational study concluded that JT distortion of CH_4_^+^ occurs within 10 ± 2 fs. Most recently, Zinchenko and co-workers^22^ combined element-specific ATAS with full-dimensional multiconfiguration time-dependent Hartree (MCTDH) quantum dynamics simulations, finding that population transfer to the lowest adiabatic surface following SFI occurs in 3.9 ± 0.4 fs. These studies established the JT relaxation time scale for *cold* methane but leave open the question: can vibrational excitation before ionization steer the cation along distinct relaxation pathways that are discernible via XTAS?

We show that selective excitation of infrared (IR)-active modes in methane biases the nuclear dynamics following pump-induced ionization to CH_4_^+^ and leaves distinct fingerprints in the carbon K-edge spectrum. Specifically, we consider excitation of the IR-active asymmetric stretching (Q_3_) and the asymmetric scissoring (Q_4_) modes, which bias the relaxation pathways shown in Figure 1. Our calculations suggest that preparing one vibrational quantum in the Q_3_ mode enhances bond-stretching dynamics, increasing sampling of the CH_3_^+^···H complex. This geometric distortion is reported in the transient spectrum by a core-to-LUMO excitation. In contrast, preparing one vibrational quantum in the Q_4_ mode enhances angular distortions along the Jahn–Teller relaxation pathway, leading to increased sampling of the C_2v_ and D_2d_ structures, which are tracked by the core-to-SOMO transition. In the early times of the dynamics, excitation of either mode also enhances trajectories that visit a planar transition state, which connects two equivalent C_2v_ structures. Subsequently, the trajectories sample geometries near the C_2v_, planar, and D_2d_ structures. Dissociation of CH_4_^+^ into CH_3_^+^ + H is observed in a small fraction of our trajectories, despite being energetically accessible. Although the methane cation does not relax into distinct products, our study shows that it can serve as a well-characterized and experimentally accessible model for probing how mode-selective excitation steers nuclear dynamics in the earliest stages of a chemical reaction. Because mode-dependent effects are subtle and occur before the formation of distinct products, we combine trajectory classification, structure-spectrum correlation analyses, and hot–cold difference spectra to connect vibrational preparation with experimentally observable signatures of early time relaxation dynamics.

**Figure 1:**
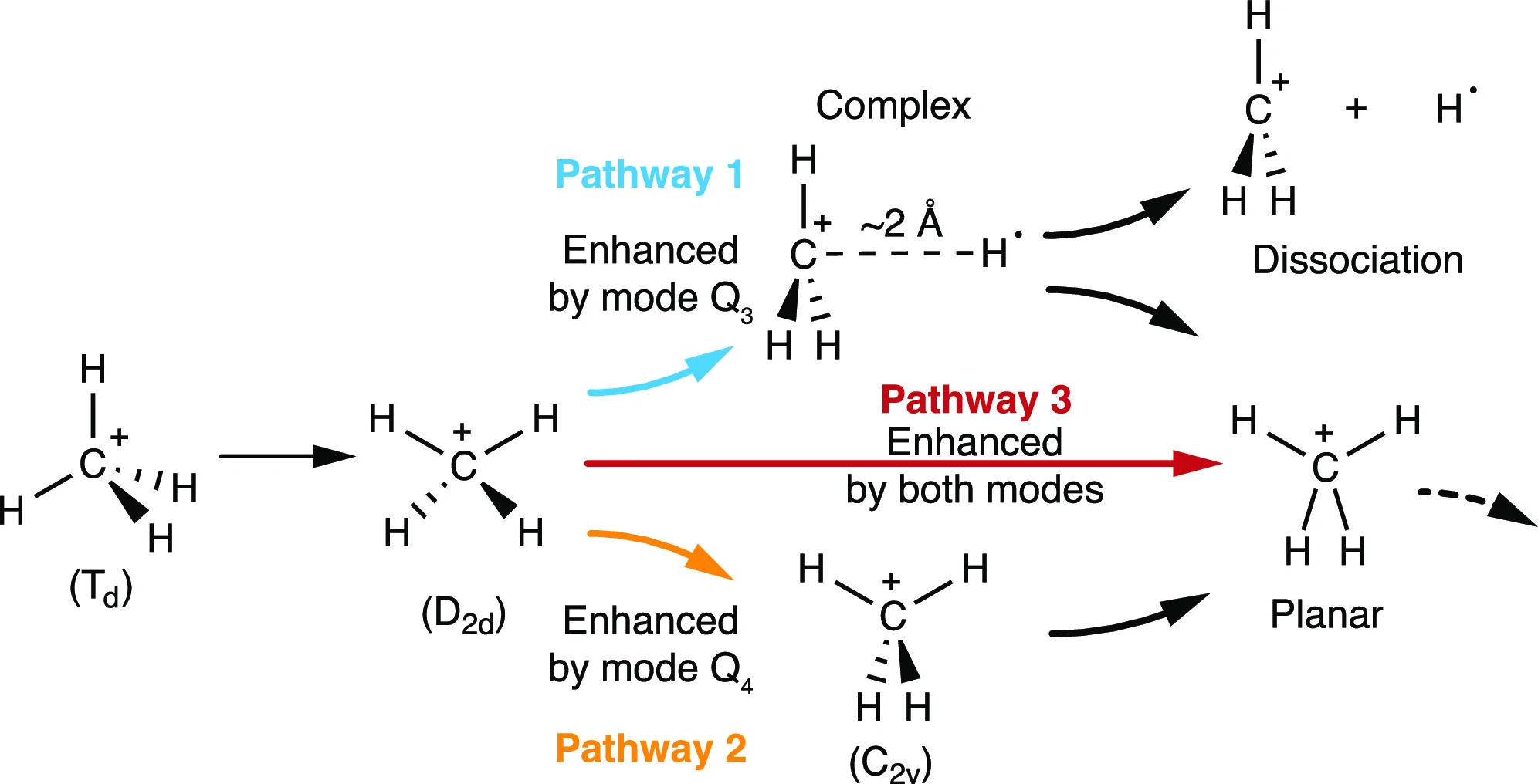
Schematic of preferential relaxation pathways induced by vibrational excitations of the asymmetric stretching (Q_3_) and the asymmetric scissoring (Q_4_) modes of methane prior to strong-field ionization.

## Electronic Structure Methodology

The dynamics of ${{CH}_{4}}^{+}$ after SFI of *hot* methane involve substantial geometric distortion. These conditions make the theoretical description of ${{CH}_{4}}^{+}$ and its carbon K-edge core-excited states particularly demanding and call for a multireference treatment of electron correlation. Our recently introduced generalized active space driven similarity renormalization group (GAS-DSRG) approach enables high-accuracy treatment of electron correlation along dissociative pathways and of transient species.^41,42^ This framework provides a complementary high-level perspective on an earlier density functional theory (DFT)-based ATAS study^23^ and helps validate the interpretation of mode-selective spectral signatures using an electronic structure approach beyond DFT.

To investigate the nuclear dynamics of CH_4_^+^ following sudden strong-field ionization from methane, we performed *ab initio* molecular dynamics (AIMD) in which the nuclear coordinates are propagated using classical equations of motion on an adiabatic potential energy surface. In this work, the forces acting on the nuclei are computed *on-the-fly* from electronic structure calculations as the dynamics evolve. The energy and analytic gradients of the methane cation were computed at the coupled cluster with singles and doubles level with unrestricted Hartree–Fock orbitals (UHF-CCSD) and a correlation-consistent polarized core double-ζ basis set (cc-pCVDZ).^43,44^ This level of theory is comparable to the ones used in previous studies that have performed AIMD trajectories on CH_4_^+^,^23^ at the EOM-IP-CCSD/cc-pVDZ level of theory. As shown in Table S2, we perform a comparison of the energy differences between the optimized *T*_d_ geometry of CH_4_ with the Jahn–Teller distorted optimized C_2v_ geometry of CH_4_^+^. This comparison shows that the energy difference at the UHF-CCSD/cc-pCVDZ level of theory (1.589 eV) is within 0.1 eV from the corresponding CCSD­(T)/cc-pCVQZ value (1.628 eV). Furthermore, as shown in Figure S1, the UHF-CCSD/cc-pCVDZ level of theory reproduces the energetics of the intrinsic reaction coordinate (IRC) of CH_4_^+^ well, giving a nonparallelity error compared to CCSD­(T)/cc-pCVQZ energies that is within 3 m*E*_h_ (ca. 2 kcal mol^–1^). These comparisons demonstrate that this level of theory provides a suitable compromise between accuracy and the cost of propagating nuclear dynamics.

We probe the nuclear dynamics and electronic structure of ${{CH}_{4}}^{+}$ after sudden ionization by computing core-excited spectra snapshots along each AIMD trajectory, using the GAS-DSRG framework.^41^ To provide an accurate description of electron correlation in core-excited states while balancing the high computational cost of performing numerous AIMD trajectories, we employ the MR-DSRG third-order perturbative approximation (DSRG-MRPT3)^45^ with a correlation-consistent polarized core triple-ζ Douglas-Kroll basis set (cc-pCVTZ-DK),^43,44,46^ treating scalar relativistic effects with X2C. This methodology has been extensively benchmarked on a test set comprising 40 organic molecules of varying sizes, yielding carbon K-edge excitation energies with a mean absolute deviation of 0.29 eV with respect to the experiment.^47^

Further details on the GAS-DSRG formalism and AIMD computations can be found in Section S1 of the Supporting Information.

## Results and Discussion

### Analysis of Molecular Dynamics Trajectories

We begin by summarizing the main features of the *ab initio* molecular dynamics simulations. First, we determine the stationary structures visited during the cation dynamics starting from cold methane to analyze the nuclear dynamics when starting from vibrationally excited methane. From the first 40 fs of our *ab initio* molecular dynamics simulations, we determine four potential stationary point structures of CH_4_^+^ which correspond to *T*_d_, C_2v_, planar, and D_2d_ geometries,^40,48^ and a nonstationary structure corresponding to the CH_3_^+^···H complex close to the second-order C_3v_ transition state.^48^ We utilize these geometries as reference structures and use a vector encoding of their geometry to represent them

$$g=\left(r_{1},...,r_{4},d_{12},d_{13},...,d_{45}\right)$$

where *r*_*i*_ is the C–H_*i*_ bond length and *d*_*ij*_ is the distance between the *i* ^th^ and *j* ^th^ hydrogen atoms. Using this representation, we determine the distance

1 $$\chi \left(g,g_{r}\right)=\sqrt{\frac{1}{4}\sum_{i=1}^{4}{\left(g_{i}-g_{r,i}\right)}^{2}+\frac{1}{6}\sum_{i=5}^{10}{\left(g_{i}-g_{r,i}\right)}^{2}}$$

of any structure visited during the dynamics (**g**) and one of the five reference structures (**g**_*r*_, *r* = 1, ..., 5), minimizing over all permutations of hydrogen indices. We then analyze our trajectories by tracking the fraction of reference structures that produces the smallest χ at each time step. The results of this analysis are shown in Figure 2. From these data, we observe that within the first 5 fs of the dynamics, the dominant structure goes from *T*_d_ to D_2d_, followed by the emergence of geometries close to the C_2v_ equilibrium conformation. This is in agreement with previous quantum dynamical studies,^38,39^ which have reported CH_4_^+^ to reach a D_2d_ structure before its C_2v_ minimum. Taking the time where the population of C_2v_ structures reaches its first peak in Figure 2, we obtain a JT time of ∼9.5 fs. At later times (10–25 fs), the dynamics sample planar-like structures, after which the C_2v_ and D_2d_ geometries dominate. Notably, our computations also show that at very early times (∼5 fs), a fraction of the trajectories sample geometries near the CH_3_^+^···H complex.

**Figure 2:**
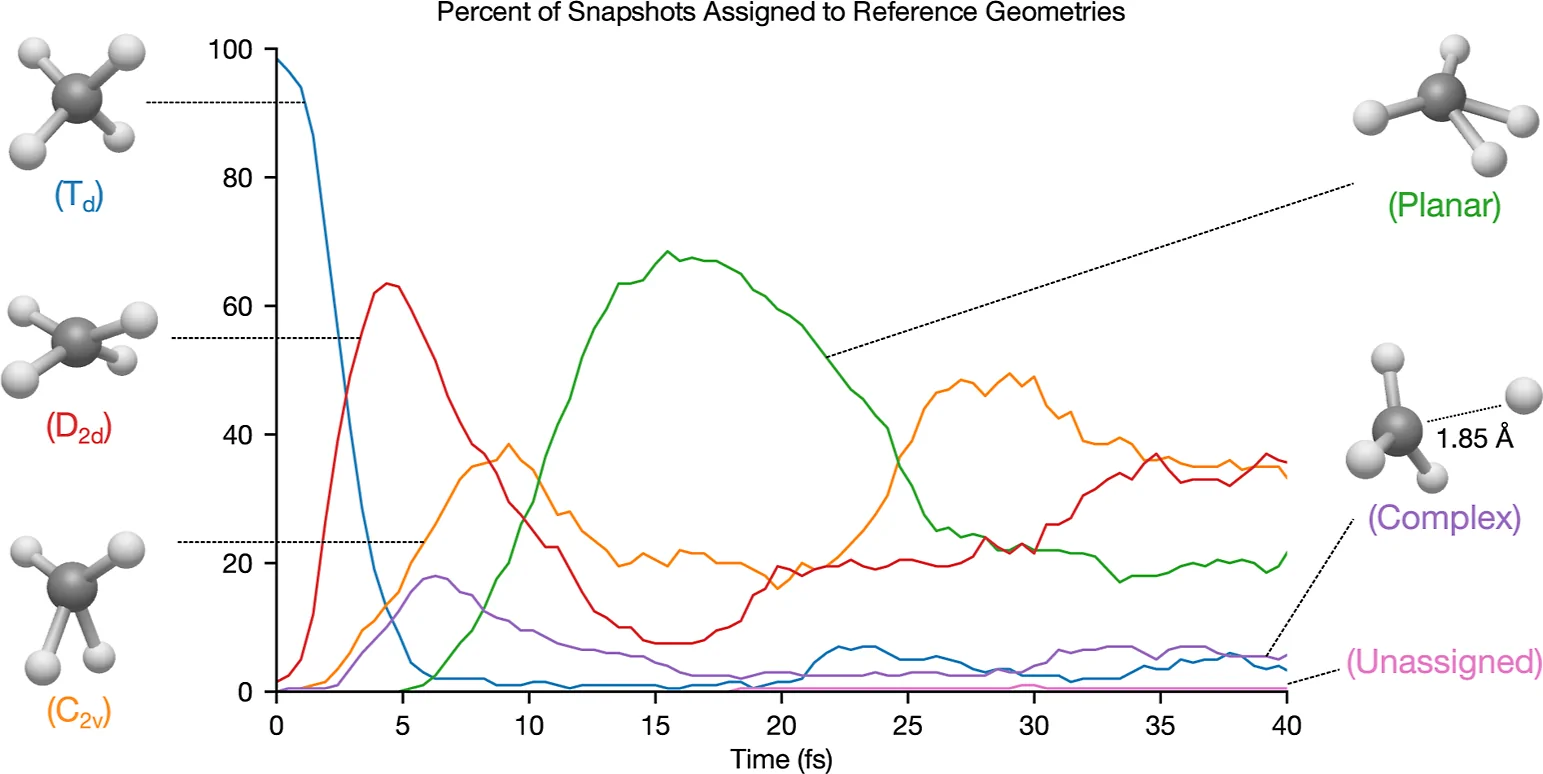
Analysis of trajectories following strong-field ionization of methane in its vibrational ground state. At each time, this plot shows the distribution of snapshots assigned to each reference geometry (*T*_d_, C_2v_, planar, D_2d_, and CH_3_^+^···H complex). A geometry along the trajectory is assigned to the reference structure with the smallest value of the distance χ [see eq 1]. Snapshots for which the χ value is greater than 0.5 Å are placed in the “Unassigned” category (less than 2% of snapshots at its maximum).

To determine how mode-selective excitation of methane affects the dynamics following SFI, we perform the assignment analysis for methane prepared with one quantum in either the *Q*_3_ or *Q*_4_ mode, subtracting the corresponding cold-methane assignment fractions. This analysis is reported in Figure 3. In this plot, positive values indicate increased sampling of a structure relative to cold methane and vice versa. The resulting traces show that vibrational excitation affects the timing of Jahn–Teller relaxation and also redirects trajectories in a mode-dependent manner. In the early stages (<10 fs), the most pronounced effect of the excitation of *Q*_3_ is the increase in the formation of the CH_3_^+^···H complex, peaking near 5 fs, with a corresponding depletion of the D_2d_ and C_2v_ components. This observation is consistent with the asymmetric stretching increasing nuclear motion along the C–H bond, diverting part of the trajectories away from the Jahn–Teller pathway in cold methane. In contrast, excitation of *Q*_4_ suppresses the complex and, in the earlier stages, increases the number of trajectories that sample C_2v_ and planar geometries, consistent with an increase in trajectories that distort the geometry along bending modes. Within the 10–30 fs time range, excitation of the *Q*_3_ mode shows a small excess of CH_3_^+^···H complex, while in the case of the *Q*_4_ mode, there is a large (∼10%) excess of trajectories near the D_2d_ structure. The fact that around 25–35 fs, the difference in population of the D_2d_ and C_2v_ structures is anticorrelated suggests that excitation of *Q*_4_ promotes trajectories that sample angular distortions instead of direct relaxation in the C_2v_ basin. Lastly, within the 30–40 fs range, *Q*_3_ returns to primarily bias the CH_3_^+^···H complex and planar CH_4_^+^ structure, while *Q*_4_ only produces an excess of the CH_4_^+^ C_2v_ structure.

**Figure 3:**
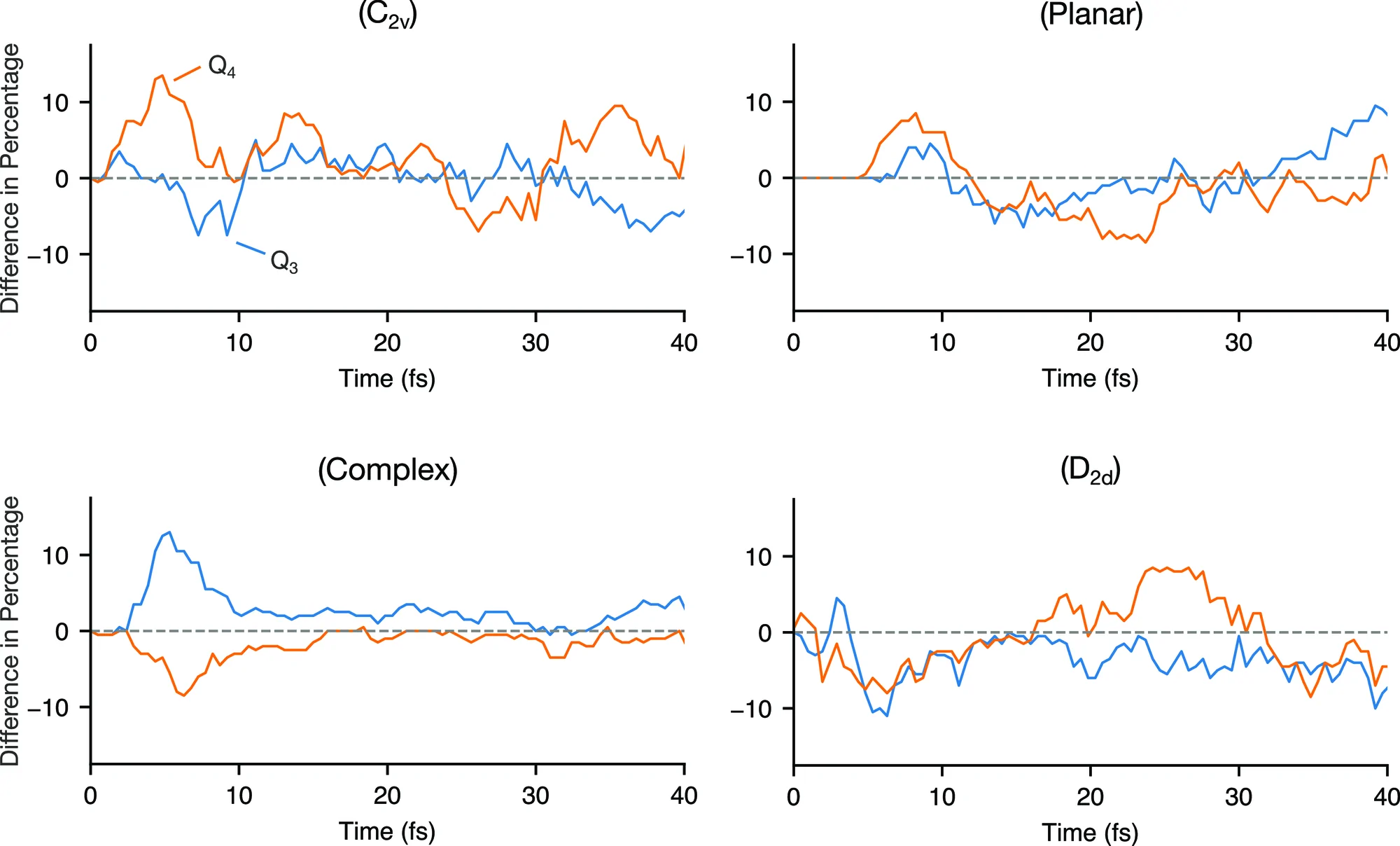
Analysis of trajectories following strong-field ionization of methane with one quantum of vibration in either the *Q*_3_ (blue curve) or *Q*_4_ (orange curve) mode. At each time, this plot shows the difference (in percentage terms) in the distribution of snapshots assigned to each reference geometry relative to the dynamics of cold methane.

Lastly, we consider how increasing the number of vibrational quanta affects the yield of the dissociated products CH_3_^+^ + H·. In cold methane, we observe no dissociative trajectories. When *Q*_3_ is excited with one quantum of vibration, a small fraction (0.5%) of trajectories break one C–H bond (defined as trajectories with one C–H distance greater than 4 Å at 40 fs). Repeating the nuclear dynamics trajectories starting with ν_3_ = 3 and 5, we observe a significant increase in the percentage of trajectories that dissociate (5.5% and 21.0%, respectively), consistent with the bond-stretching nature of the *Q*_3_ mode. In contrast, excitation of the bending mode *Q*_4_ leads to a negligible number of dissociative trajectories, with no noticeable increase in yield with the number of quanta (0.5, 0, and 1%, respectively, for ν_4_ = 1, 3, and 5). In all cases, no trajectories led to dissociation of two C–H bonds within the first 40 fs.

### Simulation of Transient Carbon K-Edge Spectra

Next, we analyze the spectral features corresponding to the dynamics of cold methane. The dominant spectral feature in the simulated X-ray absorption spectrum of methane is a single 1s → 2t_2_ transition at 289.4 eV (as predicted by GAS-DSRG). As shown in Figure 4A, sudden ionization to CH_4_^+^ removes the dominant methane transition and gives rise to two new peaks: a core-excitation to the newly partially vacant 1t_2_ orbital (277.0 eV), and a higher 1s → 2t_2_ transition at 292.0 eV. Distortion from the *T*_d_ to the JT-relaxed C_2v_ geometry^49–51^ through the D_2d_ intermediate^38,39^ blue-shifts the low-energy feature by about 4 eV and modestly shifts the higher-energy feature, reflecting the lowering of symmetry and increasing the multiconfigurational character (see Table S3). In Table S4, we analyze the evolution of the electronic structure along the bond-stretching coordinate.

**Figure 4:**
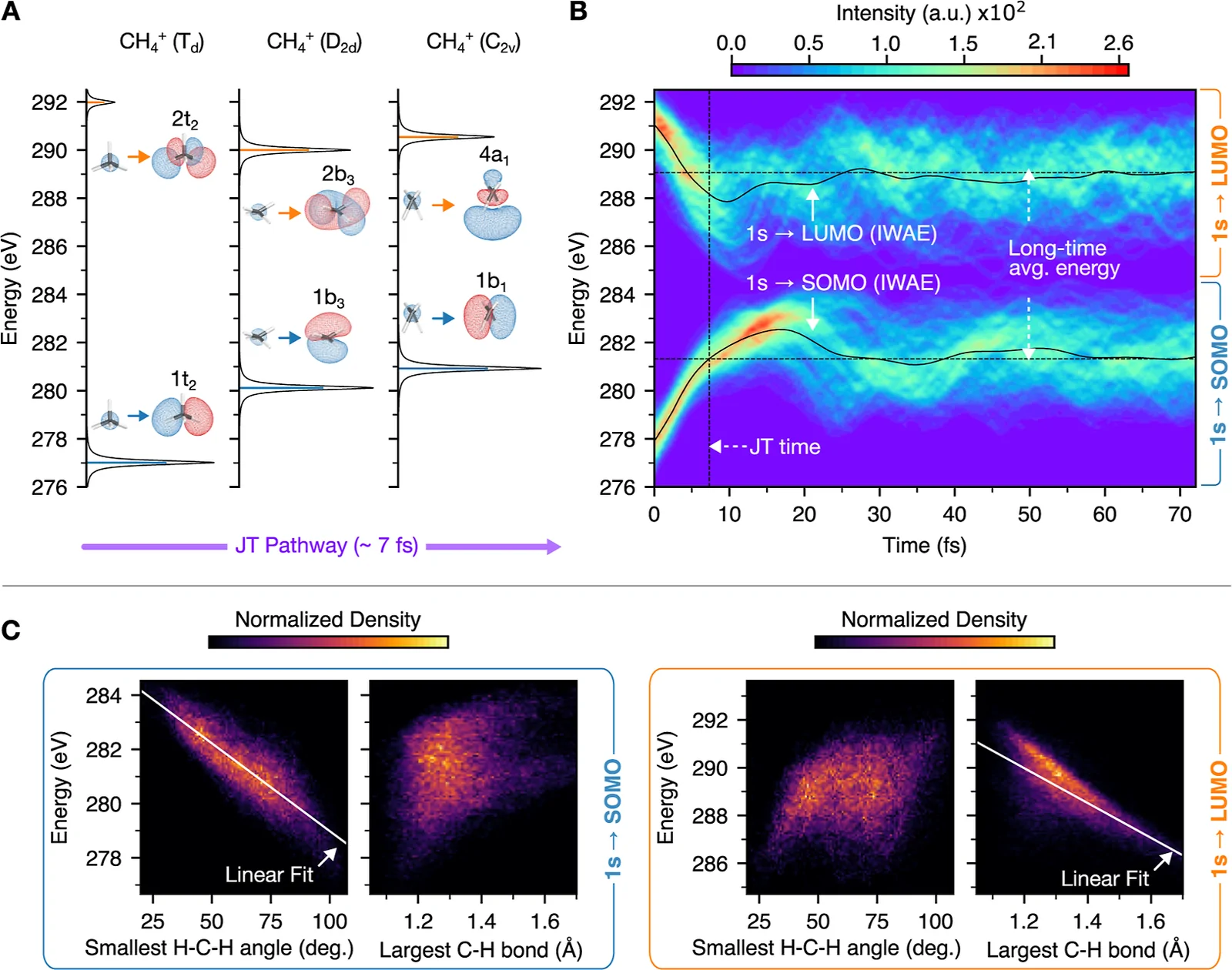
(A) Carbon K-edge spectrum of CH_4_^+^ along key structures in the Jahn–Teller relaxation pathway. (B) Simulated X-ray transient absorption spectrum of CH_4_^+^. (C) Core-excited energy vs geometry correlation plot for CH_4_^+^.

The simulated XTAS spectrum of cold methane is shown in Figure 4B. By finding the time required for the intensity-weighted average excitation energy (IWAE) of the core-to-SOMO transition to first cross over its long-time average, we arrive at an estimated spectral JT time of 7.3 fs. In ref 23 the JT time was estimated in the same way directly from the experimental time-resolved spectrum, yielding a slightly larger value (10 ± 2 fs) that is in good agreement with our value from the simulated spectrum. In contrast, the work of Zinchenko et al.^22^ reported a ∼4 fs electronic relaxation time scale from full-dimensional quantum-dynamics simulations; this quantity reflects population flow to the lowest adiabatic surface and is not directly equivalent to a JT distortion time. As observed in previous studies,^22,23,37^ the pronounced oscillations in the XTAS signal and in the IWAE are consistent with coherent nuclear motion dominated by Jahn–Teller-active scissoring/bending coordinates of CH_4_^+^ during the first ∼20 fs. At later times, as shown in Figure S3, the IWAE oscillations dampen, consistent with dephasing on an anharmonic potential energy surface.^22,23^

The core-to-LUMO signature, corresponding to the higher energy spectral feature in Figure 4B, crosses its long-time average value within the first 4.5 fs and exhibits greater dephasing by ∼8 fs. This behavior suggests that the CH_4_^+^ minimum, which is shallow near the JT manifold,^48^ maps to a wide energy range in the corresponding core-excited potential energy surface probed by the 1s → LUMO transition. To reveal the geometric dependence of the two transitions underlying the XTAS spectrum in Figure 4B, we correlate core-excitation energies with geometric variables that describe the dominant scissoring motion (the smallest H–C–H angle) and the stretching mode (the largest C–H bond) expected to be observed in the JT distortion pathway.

Notably, we observe unique geometric dependence with respect to these core-excitation energies. As shown in the left panels of Figure 4C, the core-to-SOMO excitation correlates strongly with the smallest H–C–H bond angle (left) and shows little sensitivity to C–H stretching (right). In contrast, the core-to-LUMO excitation, shown in the right panels, correlates most strongly with the largest C–H bond length. This geometric correlation is also reflected in the Fourier transforms of the IWAEs (see Figure S4).

Next, we exploit the different geometric sensitivities of the core-to-SOMO and core-to-LUMO transitions to derive experimental handles for the pathways followed after pump-ionization of vibrationally excited methane prepared in either the asymmetric stretch (*Q*_3_) or asymmetric scissoring (*Q*_4_) mode. Here, we denote the cation formed upon ionization of methane prepared in mode *Q*_*i*_ with ν_*i*_ = 1 as CH_4_^+^(ν_*i*_ = 1). Figure 5 shows the corresponding hot–cold difference spectrum (ΔmOD) constructed from the ensemble of 200 trajectories; the positive (negative) signal indicates absorption that is enhanced (suppressed) relative to the cold reference; Roman numerals denote major features in the spectrum. An analysis of the evolution of the smallest H–C–H bond angle (see Figure S5) shows that compared to the JT distortion time of cold methane (10.6 fs), excitation of *Q*_4_ reduces it (9.7 fs), while *Q*_3_ increases it (11.3 fs). Unlike our simulated spectroscopic JT distortion time (7.3 fs for cold methane), which provides an indirect measure of the structural evolution, these times are obtained directly from the dynamics data. It is important to point out that JT times derived from geometric information from nuclear dynamics and those obtained from an analysis of time-resolved spectra are inherently different and cannot be directly compared. While nuclear dynamics enable a direct analysis of the molecular structure, the corresponding JT time depends on how this quantity is defined. Likewise, time-resolved spectra provide an indirect, electronically weighted measure of the underlying geometric distortion. Because the differences in JT distortion times are subtle effects and unlikely to be resolved directly in experiments, we turn our analysis to the full hot–cold difference spectra, which provide a way to better reveal mode-dependent changes in the nuclear dynamics.

**Figure 5:**
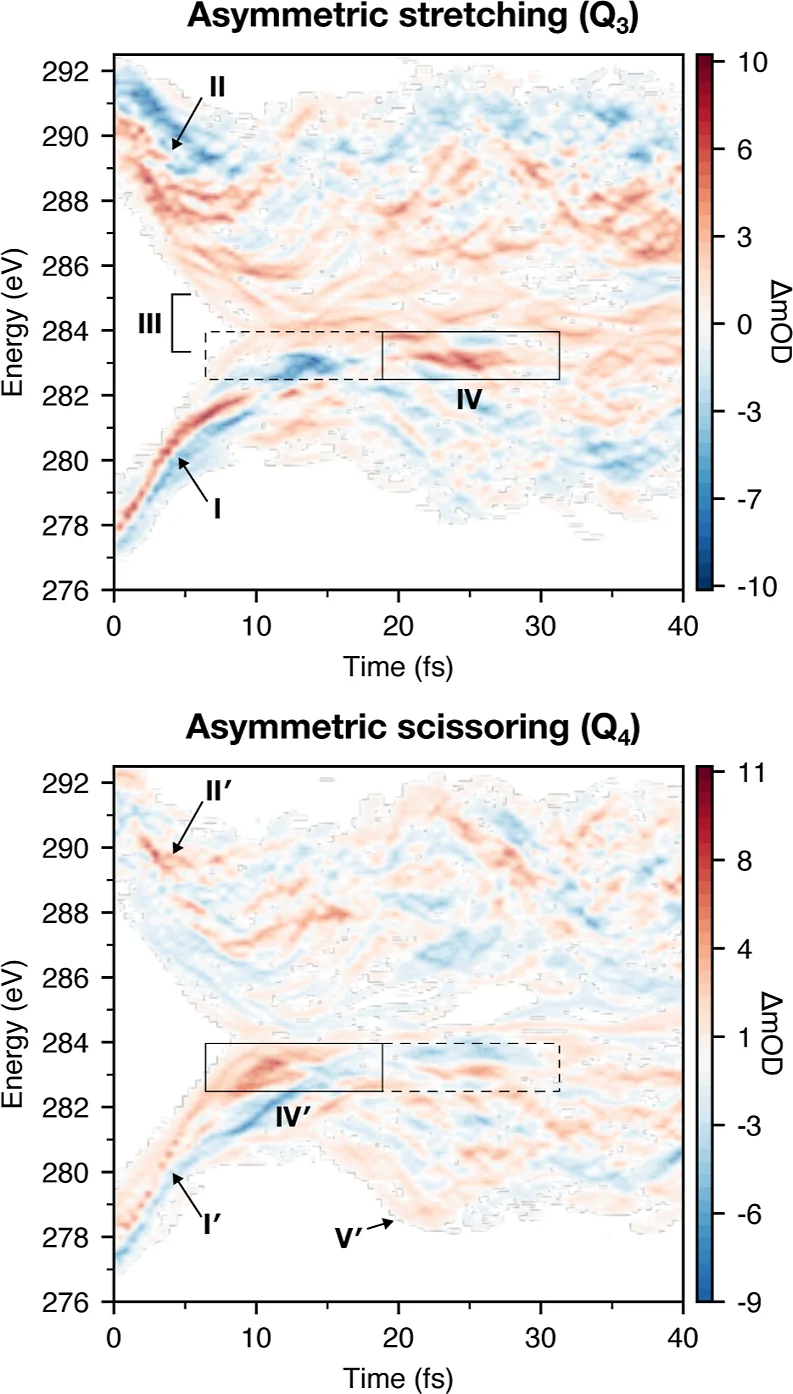
Difference X-ray transient absorption spectrum of the lowest two core-excitations of CH_4_^+^ following SFI of hot methane prepared in the ν_3_ = 1 (top) and ν_4_ = 1 (bottom) vibrational states, relative to cold methane (ν_*i*_ = 0).

Features **I** and **I** **′** in Figure 5 arise from the core-to-SOMO transition along the Jahn–Teller distortion pathway. Because the added vibrational energy broadens the initial Franck–Condon ensemble toward more distorted structures, both cases blue-shift the core-to-SOMO feature relative to the cold case. Additionally, feature **II** appears with higher contrast in the CH_4_^+^(ν_3_ = 1) difference spectrum, whereas **II** **′** is weaker and more fragmented in the CH_4_^+^(ν_4_ = 1) spectrum. Together, these trends reinforce the idea that the core-to-LUMO transition is a more sensitive reporter of bond-stretching dynamics and therefore offers stronger discrimination between the *Q*_3_-and *Q*_4_-excited pathways.

Feature **III** (Figure 5) is a long-lived signal in the XTAS difference spectrum observed only when starting from CH_4_^+^(ν_3_ = 1). To identify its origin, in Figure 6 we isolate the CH_4_^+^ XTAS contribution of Q_3_-excited trajectories whose core-to-LUMO excitation energies cross into the 283.5–285.0 eV window. The dominant contribution to feature **III** arises from trajectories exhibiting pronounced elongation of a C–H bond consistent with the CH_3_^+^···H complex configuration. This can be seen from plots of the largest C–H bond length vs time for trajectories associated with feature **III** (Figure 6), which reveal the formation of CH_3_^+^···H configurations throughout the simulated dynamics, with an average longest C–H distance of ∼1.9 Å during 7–20 fs, and one trajectory reaching a maximum separation of ∼3.0 Å at 40 fs.

**Figure 6:**
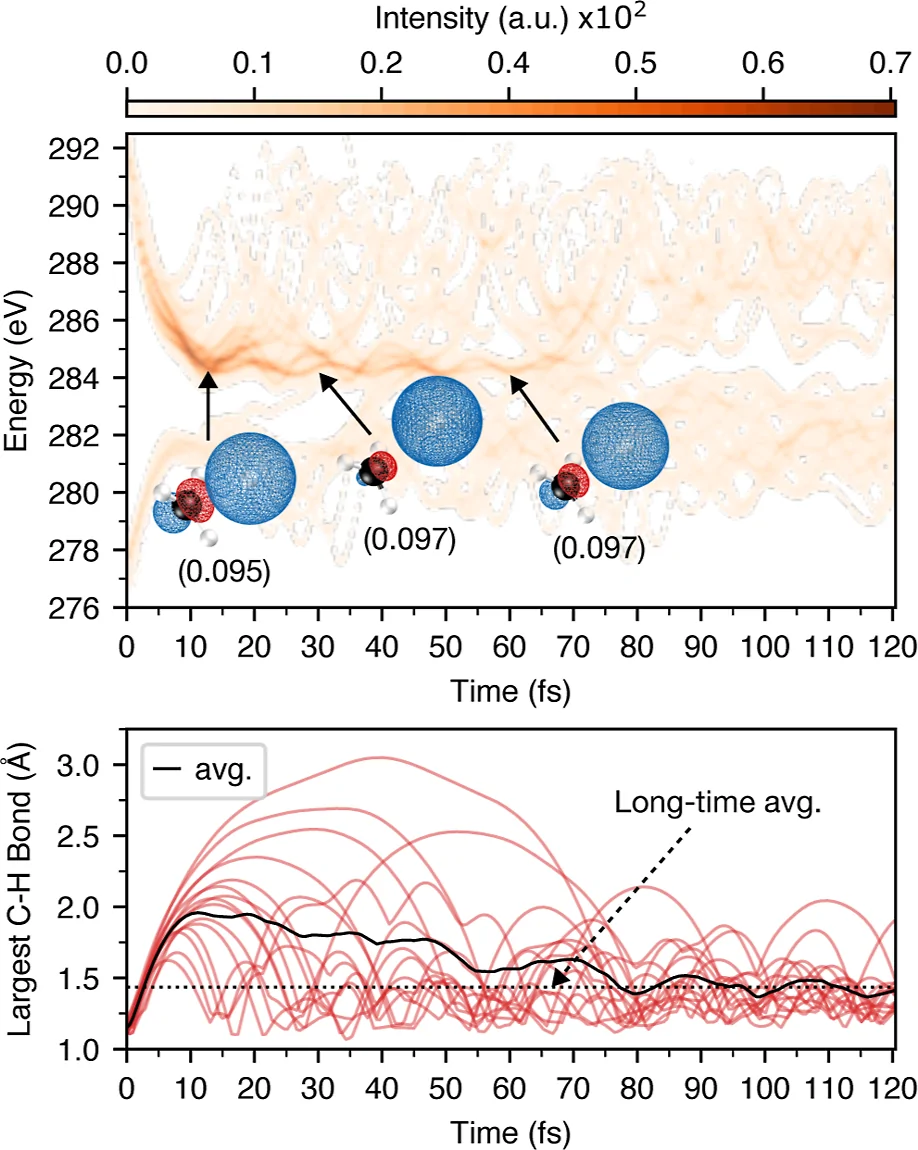
Analysis of 15 trajectories of CH_4_^+^(ν_3_ = 1) contributing to feature **III**. (Top) Carbon K-edge transient absorption spectrum with representative LUMOs for the core-to-LUMO transition and computed oscillator strengths (in parentheses). (Bottom) Largest C–H bond length vs time.

The average C–H bond length shows a trend of CH_3_^+^···H complex rebonding, which becomes more pronounced after 50 fs. Representative core-excited orbitals along the C–H bond-stretching pathway (Figure 6) show p-like density localized on the CH_3_^+^ fragment, which enhances the core-to-LUMO oscillator strength. Although these strongly elongated trajectories represent only a small fraction of the ensemble (15 out of 200 for *Q*_3_), their relatively large core-to-LUMO oscillator strengths (up to ∼0.1 in our calculations) render feature **III** visible in the difference spectrum. Because feature **III** is correlated to substantial C–H elongation, it is observed primarily for *Q*_3_ preparation, providing a direct XTAS signature of transient CH_3_^+^···H formation.

The complementary signatures of features **IV** and **IV**′ in Figure 5 are consistent with sampling of different relaxation pathways following excitations of the Q_3_ and Q_4_ modes of methane. These two features arise from core-to-SOMO excitations of CH_4_^+^ and, therefore, are observed in the low-energy region of the difference spectrum (below 284 eV). To connect these features to structural dynamics, in Figure 7, we plot the respective core-to-SOMO excitations belonging to the trajectories that contribute to these spectroscopic signatures along with representative geometries. For the Q_3_-excited trajectories, feature **IV** is preceded by a coherent set of trajectories that, at approximately 12 fs, sample geometries near the planar CH_4_^+^ transition state (see structure **a**). In this configuration, increased p-like character in the SOMO enhances the transition intensity (oscillator strength *f* ≈ 0.046 au). Later, around 25 fs, feature **IV** (see structure **b**) is reached, in which the carbon atom and three hydrogen atoms are approximately coplanar, while the fourth hydrogen atom is displaced away from the plane. This distortion leads to slightly reduced p-orbital character in the SOMO and a smaller oscillator strength (0.042 au). Next, we analyze the trajectories with a Q_4_ excited mode associated with feature **IV**′. This feature is associated with a distorted C_2v_–like structure (structure **c**), appearing around 12 fs, in which the elongated C–H bonds characteristic of the CH_4_^+^ C_2v_ structure are twisted out-of-plane relative to one another. Following the appearance of this feature, the ensemble signal dephases rapidly, reflecting the dispersion of trajectories along the anharmonic cationic potential energy surface. Lastly, at approximately 22.5 fs, feature **V**′ corresponds to a D_2d_–like structure of CH_4_^+^ (structure **d**), resulting from a fraction of the same trajectories that contribute to feature **IV**′. According to our simulations, the low core-to-SOMO transition energy of feature **V**′ (278–279 eV) makes this set of trajectories detectable in the corresponding difference spectrum in Figure 5. While the experimental observation of such features is within the reach of existing transient X-ray absorption techniques, the most challenging aspect will likely be achieving a sufficiently large population of vibrationally excited states to resolve the hot–cold difference features predicted here. Although simulating spectra under realistic experimental conditions is beyond the scope of this work, our preliminary analysis shows that the difference spectrum features in Figure 5 remain visible even under larger spectral broadening and when the starting point of trajectories is shifted randomly in time (see Figures S6 and S7).

**Figure 7:**
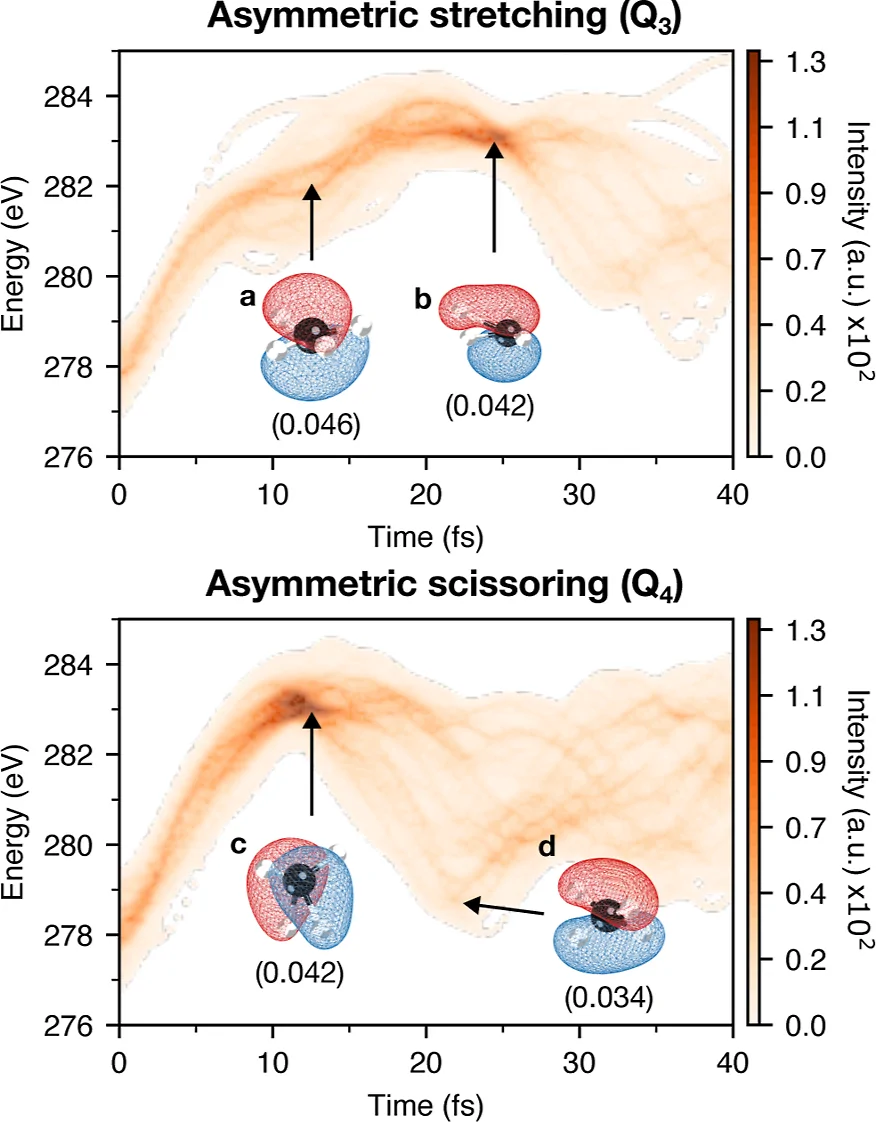
Carbon K-edge transient absorption spectrum of (top) CH_4_^+^(ν_3_ = 1) and (bottom) CH_4_^+^(ν_4_ = 1) trajectories contributing to features **IV** and **IV**′, respectively. Representative SOMOs are plotted for the core-to-SOMO transition with computed oscillator strengths given in parentheses.

## Conclusion

In conclusion, we simulated the carbon K-edge X-ray transient absorption spectrum of CH_4_^+^ following strong-field ionization of vibrationally cold and mode-excited methane. We find that the lower-energy core-to-SOMO transition primarily tracks Jahn–Teller symmetry breaking through its strong correlation with the smallest H–C–H angle, whereas the core-to-LUMO transition is most sensitive to the largest C–H bond length and therefore reports on bond-stretching and dissociation dynamics.

Taken together, our results complement and extend prior experimental and theoretical studies^22,23^ by (i) pinpointing which K-edge transitions isolate angular (JT/scissoring) versus bond-stretching motion, (ii) quantifying CH_3_^+^···H transient complex formation for vibrationally prepared methane, and (iii) providing *testable* pump–probe predictions that differentiate the distinct dynamics following preparation of the ν_3_ = 1 and ν_4_ = 1 hot states of methane via distinct early-time spectral markers and pathway-specific features in the XTAS signal. Looking ahead, the ability to discriminate pathways with specific carbon K-edge signatures opens a route to extract a more detailed description of ultrafast chemical dynamics using XTAS and highlights the value of tightly coupled theory–experiment efforts in which mode-selective pump–probe measurements are interpreted and refined with predictive simulations of both nuclear motion and core-level spectroscopy.

## Supplementary Material



## Data Availability

The data that support the findings of this study are available within the article, its Supporting Information, and from the corresponding author upon reasonable request.

## Notes

Details of the molecular dynamics protocol; details of the GAS-DSRG-MRPT3 carbon K-edge spectra calculations; computational details for the AIMD and core-excitation calculations; benchmark energies and geometries; IRC comparison of the CH_4_^+^ Jahn–Teller relaxation pathway; initial-condition energy distributions for cold and hot methane; carbon K-edge excitation energies, oscillator strengths, and CI character of CH_4_^+^ along Jahn–Teller distortion and C–H bond-stretching pathways; damping and Fourier analysis of the core-to-SOMO and core-to-LUMO intensity-weighted average energy signals; geometric and spectral signatures used to extract Jahn–Teller distortion times; effect of increased spectral broadening on the predicted hot–cold XTAS difference spectra; and the effect of timing noise on the predicted hot–cold XTAS difference spectra (PDF)
