# Supplementary material for: Discerning Mode-Controlled Relaxation Pathways in Photoionized Methane via X‑ray Transient Absorption Spectroscopy
Source: J Am Chem Soc. 2026 Aug 13;148(33):35626–34. doi: 10.1021/jacs.6c07197 (PMC13523736; doi:10.1021/jacs.6c07197)
Supplement: Supplementary file 1 [file ja6c07197_si_001.pdf]

Supplementary Information: “Discerning Mode-Controlled Relaxation Pathways in Photoionized Methane via X-ray Transient Absorption Spectroscopy"

Kevin Marin[1] and Francesco A. Evangelista[1, a)]

Department of Chemistry and Cherry Emerson Center for Scientific Computation, Emory University, Atlanta, Georgia, 30322, U.S.A.

[a)] Electronic mail: francesco.evangelista@emory.edu

S1. COMPUTATIONAL METHODS

A. *Ab initio* molecular dynamics and sampling of initial conditions

The classical propagation of the nuclear dynamics on an adiabatic potential energy surface involves solving the Hamiltonian equations of motion for the position (q_i) and conjugate momentum (p_i) of each nuclear coordinate (i). The classical equations of motion are solved using the velocity Verlet (VV) scheme. We perform multiple independent dynamic simulations (200 trajectories) with varying initial conditions (ICs). To properly account for quantum effects, including *wave packet* spreading, we employ a Wigner distribution (WD)[1] which provides a pseudo-phase-space probability distribution of positions and momenta of a given quantum state.[2,3] Given a quantum state $\Psi(x)$ of a one-dimensional system, the Wigner distribution of position (Q) and momentum (P) is given as the Fourier transform of the product between $\Psi(x')$ and $\Psi^*(x)$ with respect to the difference coordinate, $s = (x - x')$:

$$W(Q,P) = \frac{1}{(2\pi\hbar)^N} \int ds \, \langle x'|\Psi\rangle \langle\Psi|x\rangle \exp\left[-\frac{i}{\hbar}P(x - x')\right] \tag{S1}$$

where, $x = Q + s/2$ and $x' = Q - s/2$. This function recovers the quantum mechanical probability distributions for position and momentum when projected onto the P or Q axis, respectively:

$$\int W(Q,P)dP = |\Psi(Q)|^2 \tag{S2}$$

$$\int W(Q,P)dQ = |\varphi(P)|^2 \tag{S3}$$

and provides the correct expectation values of a generic physical operator $O(Q,P)$:

$$\int\int W(Q,P)O(Q,P)dQdP = \langle O\rangle \tag{S4}$$

It is important to note that since the quantum mechanical energy (E^{QM}) in this phase-space representation is recovered by averaging over a spread of classical energies ($\langle E^{\mathrm{cl}}\rangle$) with probability $W(Q,P)$, the Wigner distribution may lead to sample trajectories with unphysically large starting energies.[4] Fixed-energy sampling schemes,[5–7] or imposing a minimum threshold on the allowed values of $W(Q,P)$ (which produce large energies),[8] are common solutions to this problem. In this work, we employ the latter method, selecting a threshold of 0.01 a.u., for which the highest energy sampled remains below the dissociation limit while the average energy still accurately reproduces the correct quantum mechanical value, as shown in Fig. S2. To generate initial conditions for a

selectively-excited mode, Q_k, we use the Wigner distributions for all modes different from Q_k, while for mode Q_k, we first sample the initial mode displacement from the wavefunction probability distribution and then adjust the corresponding initial momentum by ensuring that the sum of the kinetic ($T_k = \frac{1}{2}\dot{Q}_k^2$) and potential energy ($V_k = \frac{1}{2}\sqrt{\omega_k}Q_k^2$) equals to the excited harmonic oscillator energy $\frac{3}{2}\hbar\omega_k$. In this procedure, displacements along Q_k that fall outside the classical turning points are excluded.

To build the Wigner distribution, we consider the eigenstates of the nuclear Hamiltonian in the harmonic approximation, which can be expressed as a sum of one-dimensional harmonic oscillator Hamiltonians, each corresponding to a normal mode coordinate (Q_κ):

$$\hat{H}_{\text{nuc}} = \sum_{\kappa}^{N} \left(-\frac{\hbar^2}{2} \frac{\partial^2}{\partial Q_\kappa^2} + \frac{1}{2}\omega_\kappa^2 Q_\kappa^2 \right) \tag{S5}$$

where N is the number of normal modes and ω_κ is the angular frequency of the κth vibrational mode. The corresponding wavefunction is a product of uncoupled harmonic oscillator wavefunctions (ψ_{ν_κ}):

$$\Psi_{\nu_1 \ldots \nu_N} = \prod_{\kappa}^{N} \psi_{\nu_\kappa}(Q_\kappa) \tag{S6}$$

with ν_κ representing the quantum number associated with the κ-th harmonic oscillator. This yields a ground-state vibrational wavefunction that is a product of Gaussian functions:

$$\Psi_{0\ldots 0} = \prod_{\kappa}^{N} \psi_0(Q_\kappa) = \prod_{\kappa}^{N} \left(\frac{\omega_\kappa}{\pi\hbar}\right)^{\frac{1}{4}} \exp\left[-\frac{\omega_\kappa}{2\hbar}Q_\kappa^2\right] \tag{S7}$$

To simultaneously sample positions and conjugate momenta at the zero-point energy level of the molecule, we exploit the fact that in a normal mode basis, the Wigner distribution can be expressed as a product of individual Wigner functions corresponding to each coordinate:

$$W(\{Q_1, \ldots, Q_N\}, \{P_1, \ldots, P_N\}) = \prod_{\kappa}^{N} W(Q_\kappa, P_\kappa) \tag{S8}$$

For a given vibrational state ν of the one-dimensional harmonic oscillator, analytical expressions of the individual Wigner functions are given by:

$$W^{(\nu)}(\tilde{Q}_\kappa, \tilde{P}_\kappa) = \frac{2(-1)^\nu}{\pi\hbar} L_\nu(\tilde{P}_\kappa + \tilde{Q}_\kappa) \exp\left[-2\left(\tilde{P}_\kappa^2 + \tilde{Q}_\kappa^2\right)\right] \tag{S9}$$

where $L_\nu(x)$ is a Laguerre polynomial and $\tilde{Q}_\kappa = Q_\kappa\sqrt{\omega_\kappa/\hbar}$ and $\tilde{P}_\kappa = P_\kappa/\sqrt{\omega_\kappa\hbar}$ are dimensionless analogs of the coordinates Q_κ and P_κ, respectively.

B. Evaluation of transient core-excitation spectra

In GAS-DSRG, a target electronic state $|\Psi_\alpha\rangle$ is approximated with a generalized active space self-consistent field (GASSCF) reference wavefunction:[9–11]

$$|\Psi_\alpha\rangle = \sum_I^{\mathrm{GAS}} c_I^\alpha |\Phi_I\rangle \tag{S10}$$

where $|\Phi_I\rangle$ denotes a determinant that satisfies a certain set of orbital restrictions and c_I^α is the corresponding coefficient. In GASSCF, both the orbital and determinant coefficients are self-consistently variationally optimized. Separate computations are performed to calculate the ground and core-excited states of CH_4^+. This is necessary to incorporate orbital relaxation (rehybridization) effects resulting from core vacancies, which significantly contribute to core excitation energies and differ substantially in the ground and core-excited states.[12–15] In both cases, the active space is partitioned into two subspaces: GAS1, which contains the carbon 1s core orbital, and GAS2, which includes four occupied and four unoccupied valence orbitals corresponding to carbon 2s and 2p and hydrogen 1s atomic orbitals (full valence). To target the ground state, the occupations of the GAS1 and GAS2 spaces are set to 2 and 7 electrons, respectively. To target core excitations, the GAS1 occupation is restricted to one electron, while that of GAS2 is set to eight electrons (seven valence electrons plus one additional electron promoted from the core). This GASSCF(1e,1o;8e,8o) reference captures static correlation due to valence orbitals and defines the relevant determinants contributing to the low-lying core-excited states of CH_4^+. To compute multiple core-excited states, we employ the state-averaged GASSCF (SA-GASSCF) formalism. Details on the choice of weights for each state are provided in Table S3.

Dynamical electron correlation is included by constructing an effective Hamiltonian, $\bar{H}$, through the DSRG formalism truncated to third order in perturbation theory. Here we provide a concise description of the main features of the DSRG. For an in-depth description of this theory, we refer the reader to refs. 16,17. In the DSRG formalism, the Hamiltonian is brought to a diagonal form by a unitary transformation written in exponential form:

$$H \mapsto \bar{H} = e^{-\hat{A}}\hat{H}e^{\hat{A}} \tag{S11}$$

where $\hat{A}$ is anti-Hermitian and can be expressed in terms of coupled-cluster operators, $\hat{A} = \hat{T} - \hat{T}^\dagger$. The goal of the DSRG transformation is to remove from $\bar{H}$ those terms that couple the reference state to its internally-contracted excited configurations, which results in dressing the remaining

terms in the Hamiltonian with dynamical correlation effects. The DSRG-MRPT3 method is obtained by expressing $\bar{H}$ as a series via the Baker-Campbell-Hausdorff (BCH) expansion, with commutators truncated to one- and two-body operators, including terms up to third-order in perturbation theory. The DSRG-MRPT3 approach is derived using as the zeroth-order Hamiltonian the average Fock matrix, resulting in a computational scheme that is size consistent and scales as $O(N_C^2 N_V^4) + O(N_A^6 N_V)$, where N_C, N_A, and N_V are the number of core (inactive doubly occupied), active (sum of all GAS orbitals), and virtual (inactive empty) orbitals.[18]

To avoid problems due to small denominators that arise in solving the equation that determine the $\hat{A}$ operator,[19–21] the DSRG approach introduces a regularization term that depends on a real parameter s. With the introduction of this parameter, all quantities that enter in the definition of the DSRG-MRPT3 method, including $\hat{A}$ and $\bar{H}$ become implicitly dependent on s. This parameter determines an energetic cutoff for the energy denominators as follows. Once s is chosen, excited configurations with corresponding energy denominator smaller than the cutoff energy $\Lambda = s^{-1/2}$ are suppressed, avoiding divergences in the correlation energy. In this work, we employ the value $s = 0.5\ E_\mathrm{h}^{-2}$ which provides a balance between the inclusion of correlation effects and avoidance of intruder states.[18,22,23]

Once the DSRG effective Hamiltonian is formed, the energies of the target states are obtained from the following eigenvalue equation

$$\bar{H}(s) \left|\tilde{\Psi}_\alpha\right\rangle = \tilde{E}_\alpha(s) \left|\tilde{\Psi}_\alpha\right\rangle \tag{S12}$$

where $\tilde{E}_\alpha(s)$ is the energy of the α-th state and $|\tilde{\Psi}_\alpha\rangle$ is a GASCI state with determinant coefficients $\tilde{c}^\alpha$ relaxed under the presence of dynamical electron correlation:

$$\left|\tilde{\Psi}_\alpha\right\rangle = \sum_I^{\mathrm{GAS}} \tilde{c}_I^\alpha \left|\Phi_I\right\rangle \tag{S13}$$

Following the approach adopted in ref. 22, the core-excitation energy ($\tilde{\omega}_{\alpha\leftarrow 0}$) for the transition between the ground state (0) and the α-th core excited state is evaluated as the energy difference between separate SA-GASSCF computations on the ground (gs) and core excited states (ex):

$$\tilde{\omega}_{\alpha\leftarrow 0} = \tilde{E}_\alpha^{\mathrm{ex}}(s) - \tilde{E}_0^{\mathrm{gs}}(s) \tag{S14}$$

We approximate the transition dipole moment by including the ground state in the core-excited state SA-GASSCF computation, with occupation restrictions consistent with a fully occupied GAS1

space (core orbitals) and zero weight ($w = 0$). Then the transition dipole moment is calculated using the relaxed wavefunctions of both the ground state and the core-excited state, as follows:

$$\vec{\mu}_{\alpha\leftarrow 0} = \sum_{pq} \vec{\mu}_{pq} \langle \tilde{\Psi}_0^{\text{ex}}(s) | \hat{a}_q^p | \tilde{\Psi}_\alpha^{\text{ex}}(s) \rangle \tag{S15}$$

where $\vec{\mu}_{pq} = -\langle \phi_p | r | \phi_q \rangle$ are the transition dipole moment integrals over molecular orbitals. It is important to note that although the ground state wavefunction here utilizes orbitals optimized for the core-excited state(s), this approach captures the leading contributions to the transition dipole moment.[22] The corresponding oscillator strength, ($f_{\alpha\leftarrow 0}$), can then be computed from the transition dipole moment as:

$$f_{\alpha\leftarrow 0} = \frac{2}{3} \tilde{\omega}_{\alpha\leftarrow 0} \left\| \vec{\mu}_{\alpha\leftarrow 0} \right\|^2 \tag{S16}$$

S2. COMPUTATIONAL DETAILS

All core-excitations are computed with the Forte[24] implementation of the DSRG-MRPT3 using a GASSCF(1e,1o;8e,8o) reference (two spaces of 1 and 8 orbitals, with 1 and 8 electrons) with a correlation-consistent polarized core triple zeta Douglas-Kroll (cc-pCVTZ-DK) basis set[25–27] treating scalar relativistic effects at the spin-free one-electron X2C level.[28–30] All geometries, unless otherwise stated, are optimized at the coupled cluster with singles and doubles level using unrestricted Hartree–Fock orbitals (UHF-CCSD) and the cc-pCVDZ basis set using the Psi4 software package.[31]

S3. CALIBRATION OF THE POTENTIAL ENERGY SURFACE USED TO PROPAGATE AIMD TRAJECTORIES

TABLE S1. Ground-state energies (E_h) of methane cation computed using UHF-CCSD and UHF-CCSD(T) theories and correlation-consistent core–valence basis sets (cc-pCVXZ, where X = D, T, or Q). Calculations were performed at two molecular geometries, one with C_{2v} symmetry and the other with T_d symmetry, with each geometry optimized at each corresponding level of theory. For T_d geometries, the methane cation energy was evaluated at the geometry optimized for the corresponding neutral molecule.

	UHF-CCSD			UHF-CCSD(T)		
	cc-pCVDZ	cc-pCVTZ	cc-pCVQZ	cc-pCVDZ	cc-pCVTZ	cc-pCVQZ
C_{2v}	-39.963118	-40.015830	-40.030375	-39.966384	-40.020994	-40.036034
T_d	-39.904734	-39.955507	-39.970168	-39.908181	-39.961047	-39.976206
$\lvert\Delta E\rvert$	0.058384	0.060324	0.060207	0.058204	0.059947	0.059828

TABLE S2. Geometric parameters of CH_4, CH_4^+, and CH_3^+ at their respective T_d, C_{2v}, and C_{3v} equilibrium structures, optimized at the UHF-CCSD/cc-pCVDZ and UHF-CCSD(T)/cc-pCVQZ levels of theories. Bond lengths r_n (Å) and bond angles θ_n (°) are reported in ascending order.

Parameters	r_1	r_2	r_3	r_4	θ_1	θ_2	θ_3	θ_4	θ_5	θ_6
CH_4 (T_d)										
CCSD/cc-pCVDZ	1.101	1.101	1.101	1.101	109.46	109.47	109.47	109.47	109.48	109.49
CCSD(T)/cc-pCVQZ	1.086	1.086	1.086	1.086	109.46	109.47	109.47	109.47	109.47	109.48
CH_4^+ (C_{2v})										
CCSD/cc-pCVDZ	1.096	1.096	1.194	1.194	55.97	113.86	113.87	114.1	114.11	125.18
CCSD(T)/cc-pCVQZ	1.08	1.08	1.183	1.183	55.1	113.86	113.86	114.03	114.03	125.51
CH_3^+ (C_{3v})										
CCSD/cc-pCVDZ	1.103	1.103	1.103	–	120	120	120	–	–	–
CCSD(T)/cc-pCVQZ	1.088	1.088	1.088	–	120	120	120	–	–	–

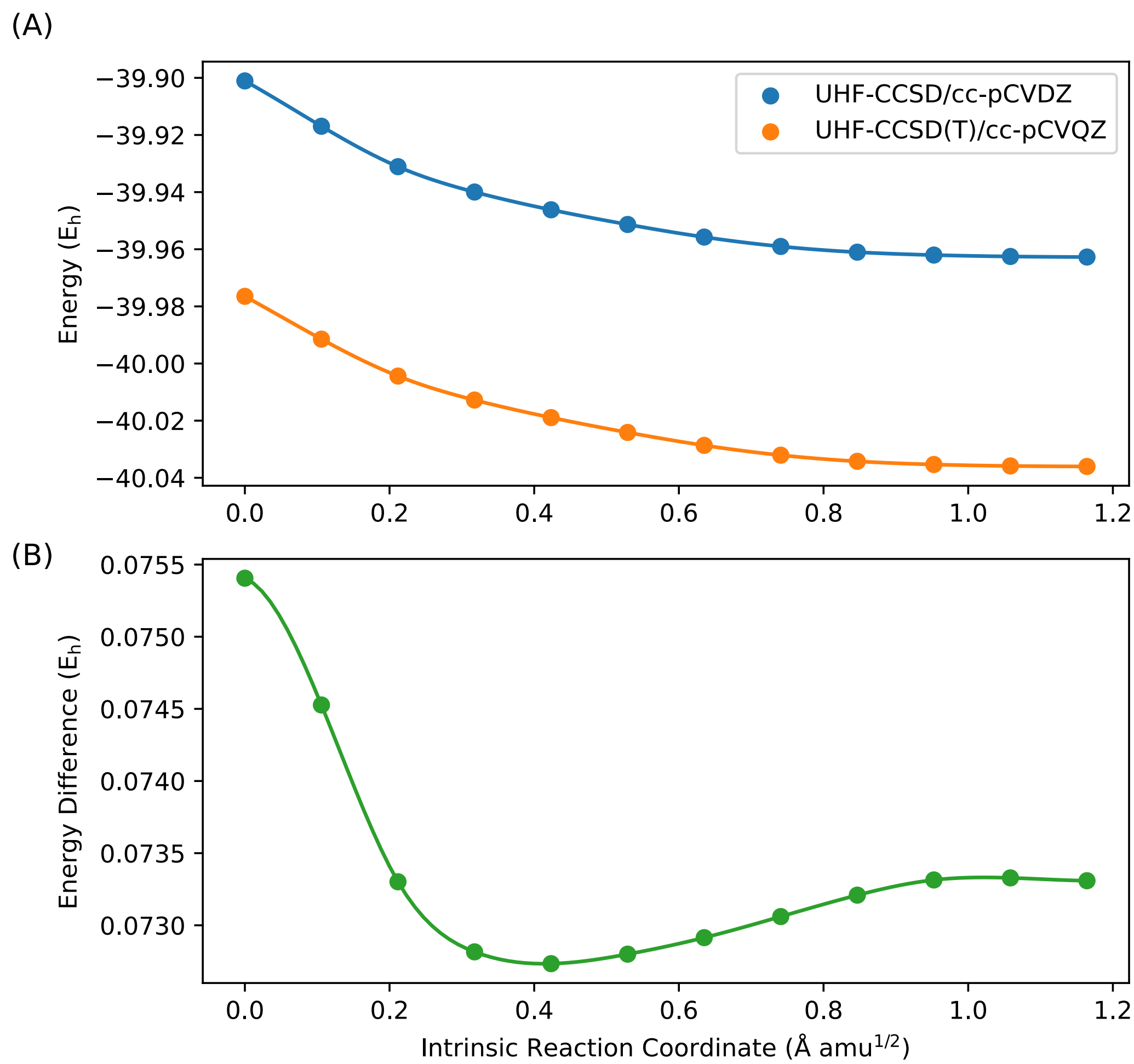

FIG. S1. (A) Single-point energy calculations along IRC relaxation pathway of methane cation from T_d to C_{2v} structure, computed with the UHF-CCSD/cc-pCVDZ (blue) and UHF-CCSD(T)/cc-pCVQZ (orange) level of theory. (B) Single-point energy difference between UHF-CCSD/cc-pCVDZ and UHF-CCSD(T)/cc-pCVQZ computed in (A). IRC is computed with the UHF-CCSD(T)/cc-pCVTZ level of theory.

S4. SAMPLING OF AIMD INITIAL CONDITIONS

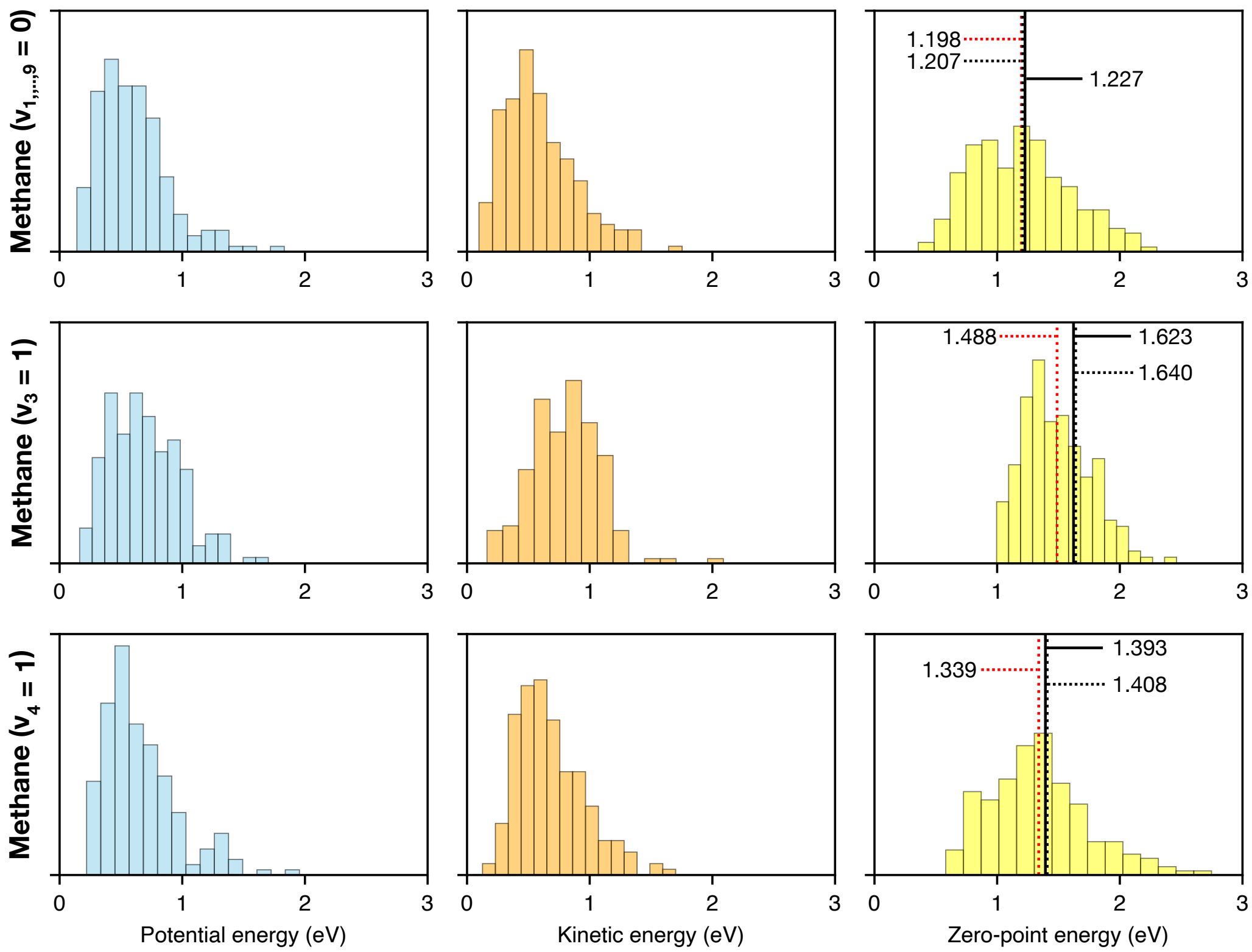

FIG. S2. Potential, Kinetic, and energy distribution of 200 initial conditions (position and velocity) obtained via Monte Carlo sampling of methane's nine normal modes (computed at the CCSD/cc-pCVDZ level of theory) and used in AIMD computations of CH_4^+ following the strong-field ionization of methane in its (top) cold (middle) $CH_4^+(\nu_3 = 1)$, and (bottom) $CH_4^+(\nu_4 = 1)$ dynamics. Quantum mechanical (solid black line), semi-classical sampled with no threshold (dotted black line) and with threshold (dotted red line) energies are indicated for the energy distributions.

S5. DETAILS OF CH_4^+ SPECTRUM ALONG KEY STRUCTURES IN JAHN-TELLER DISTORTION AND BOND DISSOCIATION PATHWAYS

TABLE S3. Carbon core-excitation energies (E) and oscillator strengths (f) of CH_4^+ at three geometries along the Jahn–Teller distortion pathway. Geometries are optimized at the UHF-CCSD/cc-pCVDZ level of theory. Peaks are computed using the DSRG-MRPT3/cc-pCVTZ-DK with a GASSCF(1e,1o; 8e,8o) reference and an X2C treatment of scalar relativistic effects. Peaks obtained using a state-specific or state-averaged formalism are labeled SS and SA, respectively. Configuration interaction (CI) weights and configurations are reported for determinants with corresponding coefficient C such that $C^2 > 15\%$.

Transition	CI Weight (%)	CI Configuration	E (eV)	f (a.u.)	Calc. Type
CH_4^+ (T_d)					
C(1s)→ $1t_2$	94.57	$(1s)^{\alpha}(1t_2)^2$	277.01	0.0318	SS
C(1s)→ $2t_2$.	24.53	$(1s)^{\beta}(1t_2)^{\alpha}(2t_2)^{\alpha}$	291.98	0.0071	SA
	24.42	$(1s)^{\beta}(1t_2)^{\alpha}(2t_2)^{\alpha}$			
C(1s)→ $2a_2$	29.64	$(1s)^{\beta}(1t_2)^{\alpha}(2a_2)^{\alpha}$	292.24	0.0033	SA
C(1s)→ $2a_2$.	29.64	$(1s)^{\beta}(1t_2)^{\alpha}(2a_2)^{\alpha}$	292.24	0.0077	SA
CH_4^+ (D_{2d})					
C(1s)→ $1b_3$	93.80	$(1s)^{\alpha}(1b_3)^2$	280.12	0.0335	SS
C(1s)→ $3a$	48.07	$(1s)^{\alpha}(1b_3)^{\alpha}(3a)^{\beta}$	290.00	0.0278	SS
	42.62	$(1s)^{\beta}(1b_3)^{\alpha}(3a)^{\alpha}$			
C(1s)→ $1b_3$, $3a$	62.55	$(1s)^{\alpha}(1b_3)^{\beta}(3a)^{\alpha}$	291.52	0.0010	SS
	18.76	$(1s)^{\beta}(1b_3)^{\alpha}(3a)^{\alpha}$			
C(1s)→ $3a$, $2b_3$	42.12	$(1s)^{\beta}(1b_2)^{\alpha}(1b_3)^2(3a)^{\alpha}$	293.53	0.0270	SS
	18.92	$(1s)^{\beta}(1b_3)^{\alpha}(2b_3)^{\alpha}$			
CH_4^+ (C_{2v})					
C(1s)→ $1b_1$	93.70	$(1s)^{\alpha}(1b_1)^2$	280.92	0.0357	SS
C(1s)→ $1b_1$, $4a_1$	63.21	$(1s)^{\alpha}(1b_1)^{\alpha}(4a_1)^{\beta}$	290.54	0.0239	SS
	20.57	$(1s)^{\alpha}(1b_1)^{\beta}(4a_1)^{\alpha}$			
C(1s)→ $1b_1$, $4a_1$	57.55	$(1s)^{\beta}(1b_1)^{\alpha}(4a_1)^{\alpha}$	291.22	0.0215	SA
	35.95	$(1s)^{\alpha}(1b_1)^{\beta}(4a_1)^{\alpha}$			
C(1s)→ $5a_1$	53.72	$(1s)^{\beta}(1b_1)^{\alpha}(5a_1)^{\alpha}$	291.91	0.0422	SA
	33.75	$(1s)^{\alpha}(1b_1)^{\alpha}(5a_1)^{\beta}$			

TABLE S4. Carbon core-excitation energies (E) and oscillator strengths (f) of CH_4^+ at three geometries along a single bond breaking pathway. Geometries are optimized at the UHF-CCSD/cc-pCVDZ level of theory, constraining a C−H bond at a fixed length (r_{CH}). Peaks are computed using the DSRG-MRPT3/cc-pCVTZ-DK with a GASSCF(1e,1o; 8e,8o) reference and an X2C treatment of scalar relativistic effects. Peaks obtained using a state-specific or state-averaged formalism are labeled SS and SA, respectively. Configuration interaction (CI) weights and configurations are reported for determinants with corresponding coefficient C such that $C^2 > 15\%$.

Transition	CI Weight (%)	CI Configuration	E (eV)	f (a.u.)	Calc. Type
CH_4^+ (r_{CH} = 1.27 Å)					
C(1s)→ $5a$	93.38	$(1s)^\alpha(5a)^2$	281.20	0.0354	SS
C(1s)→ $5a$, $6a$	62.62	$(1s)^\alpha(5a)^\alpha(6a)^\beta$	290.20	0.0296	SS
	18.31	$(1s)^\alpha(5a)^\beta(6a)^\alpha$			
C(1s)→ $5a$, $6a$	43.04	$(1s)^\alpha(5a)^\beta(6a)^\alpha$	290.58	0.0187	SA
	42.64	$(1s)^\beta(5a)^\alpha(6a)^\alpha$			
C(1s)→ $7a$	43.19	$(1s)^\beta(5a)^\alpha(7a)^\alpha$	291.89	0.0371	SA
	32.06	$(1s)^\alpha(5a)^\alpha(7a)^\beta$			
CH_4^+ (r_{CH} = 1.85 Å)					
C(1s)→ $5a$	85.34	$(1s)^\alpha(5a)^2$	281.17	0.0144	SS
C(1s)→ $5a$, $6a$	61.66	$(1s)^\beta(5a)^\alpha(6a)^\alpha$	285.66	0.0832	SS
	15.49	$(1s)^\alpha(5a)^\beta(6a)^\alpha$			
	15.34	$(1s)^\alpha(5a)^\alpha(6a)^\beta$			
C(1s)→ $5a$, $6a$	34.23	$(1s)^\alpha(5a)^\beta(6a)^\alpha$	288.96	0.0003	SA
	34.09	$(1s)^\alpha(5a)^2$			
	25.47	$(1s)^\alpha(5a)^\alpha(6a)^\beta$			
C(1s)→ $4a$, $6a$	63.66	$(1s)^\alpha(4a)^\alpha(5a)^2(6a)^\beta$	290.50	0.0000	SA
	19.17	$(1s)^\alpha(4a)^\beta(5a)^2(6a)^\alpha$			
CH_4^+ (r_{CH} = 2.43 Å)					
C(1s)→ $5a$, $6a$	54.90	$(1s)^\alpha(5a)^2$	281.86	0.0021	SS
	17.45	$(1s)^\alpha(5a)^\alpha(6a)^\beta$			
C(1s)→ $5a$, $6a$	55.38	$(1s)^\beta(5a)^\alpha(6a)^\alpha$	284.56	0.0957	SS
	26.07	$(1s)^\alpha(5a)^\beta(6a)^\alpha$			
C(1s)→ $5a$	68.88	$(1s)^\alpha(5a)^2$	286.84	0.0028	SA
	15.81	$(1s)^\alpha(5a)^\beta(6a)^\alpha$			
C(1s)→ $4a$, $6a$	63.74	$(1s)^\alpha(4a)^\alpha(5a)^2(6a)^\beta$	289.29	0.0000	SA
	16.92	$(1s)^\alpha(4a)^\beta(5a)^2(6a)^\alpha$			

S6. DEPHASING AND COHERENCE IN XTAS SPECTRA OF COLD CH_4^+

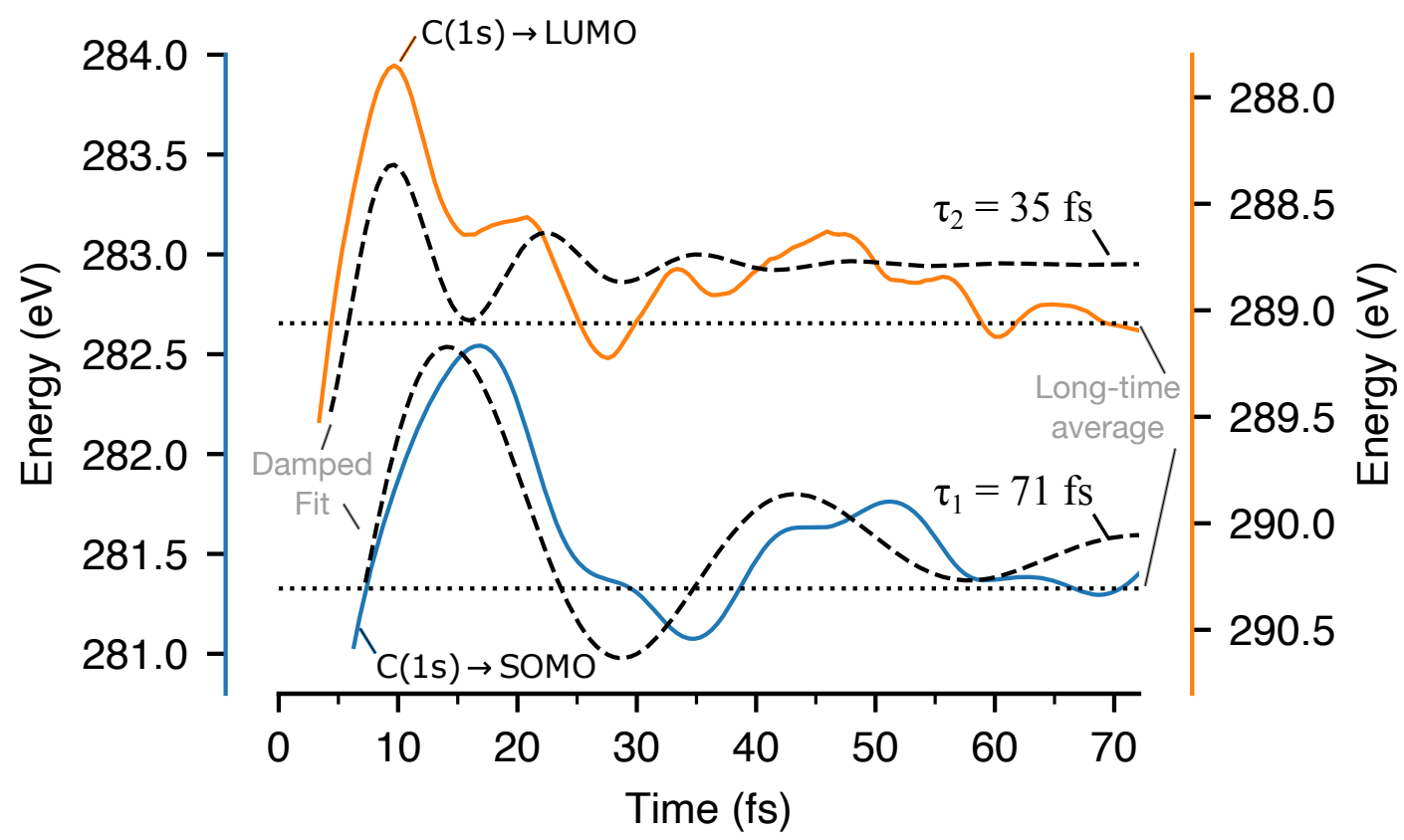

FIG. S3. Dampened harmonic oscillator fit of the C1s → SOMO IWAE signal with $\omega_1 = 1025$ cm^{-1} resulting in a dampening lifetime $\tau_1 = 71$ fs, and C1s → LUMO IWAE signal with $\omega_2 = 2600$ cm^{-1} resulting in a dampening lifetime $\tau_2 = 35$ fs.

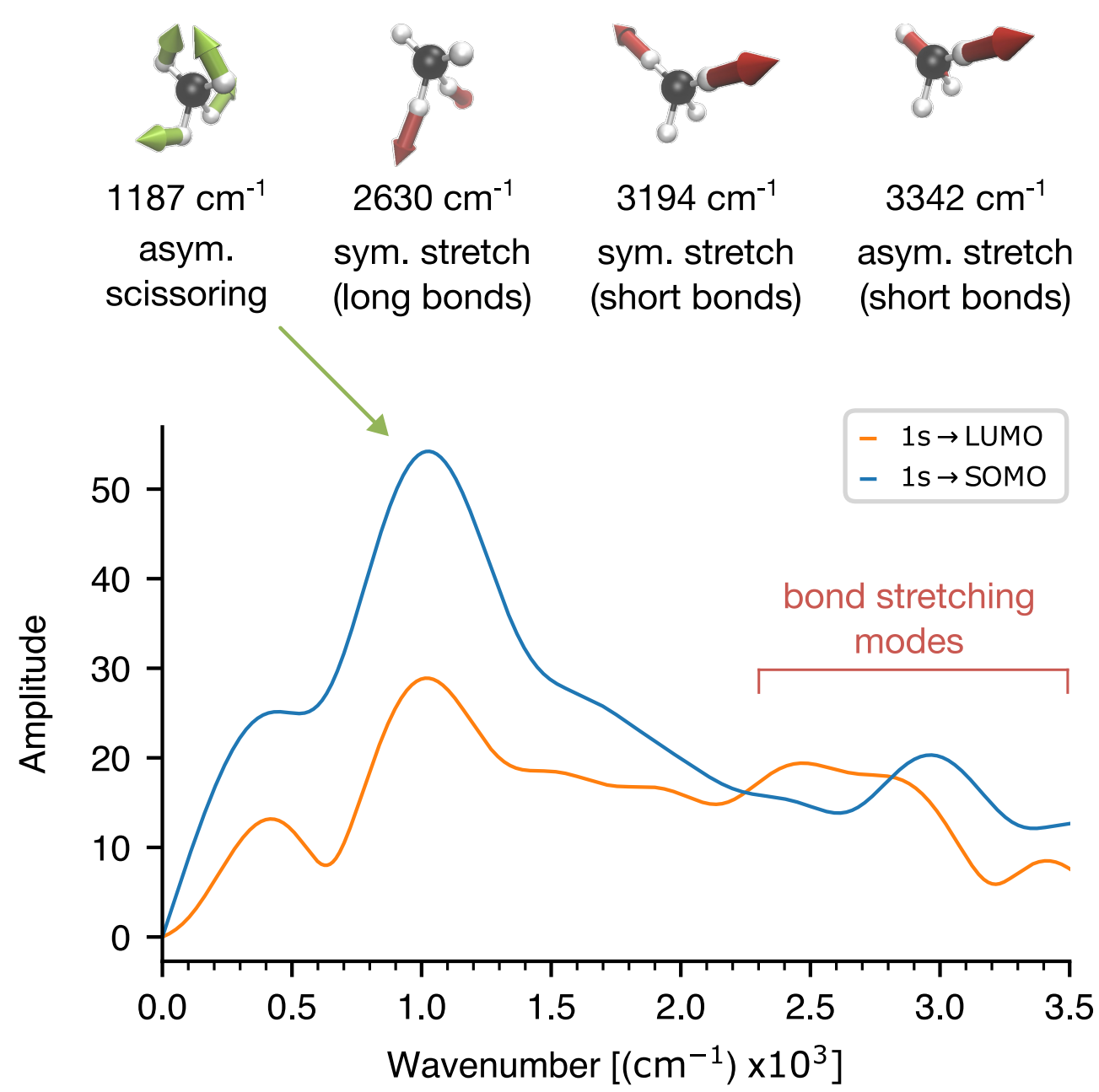

FIG. S4. Fourier transforms of the 1s → SOMO and 1s → LUMO intensity-weighted average energy signals and respective normal modes of CH_4^+ at C_{2v} symmetry.

S7. JAHN-TELLER DISTORTION SIGNATURE IN XTAS SPECTRA

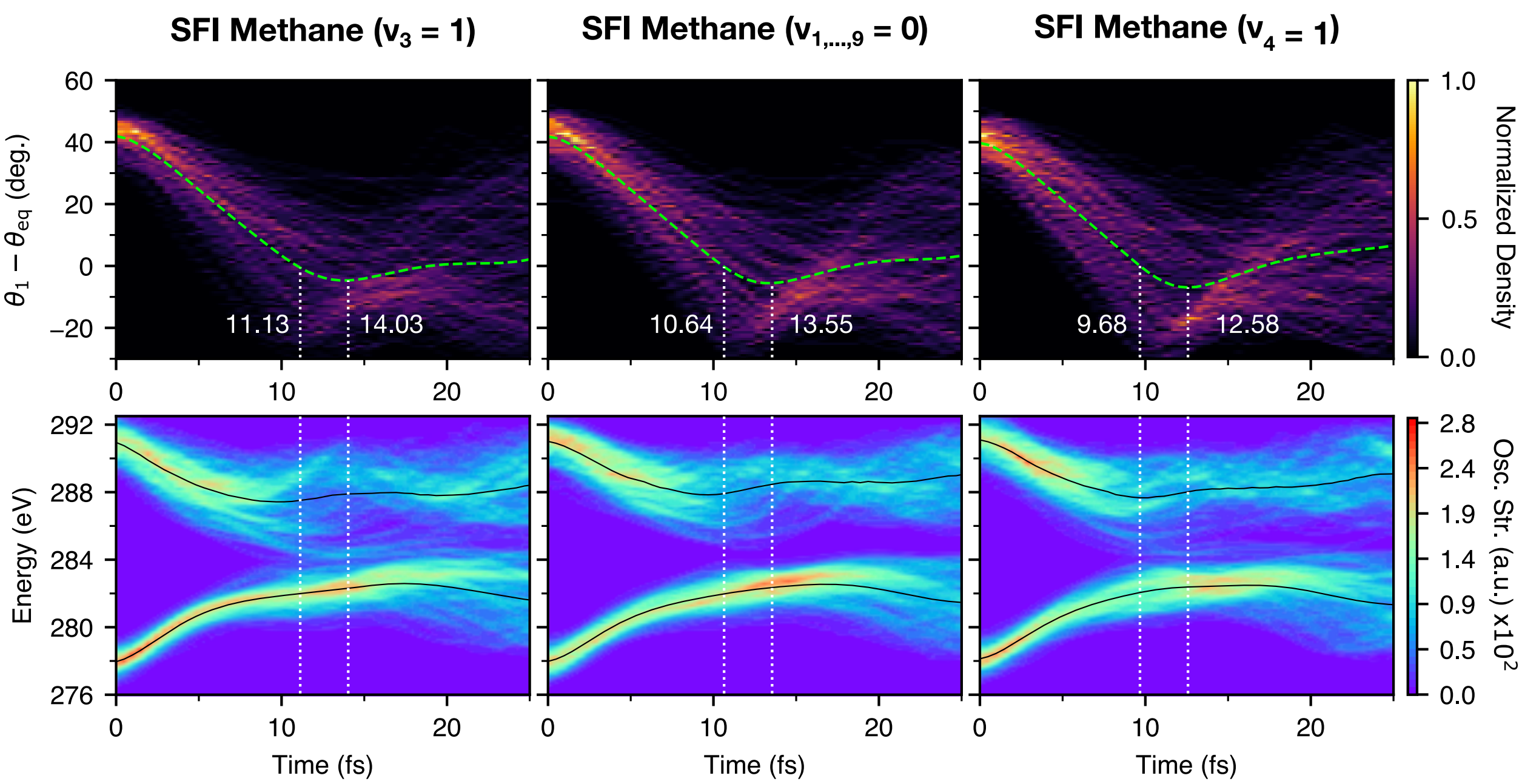

FIG. S5. (Top row) difference between the smallest H−C−H bond angle (θ_1) and the smallest H−C−H bond angle in the $CH_4^+(C_{2v})$ equilibrium geometry along all 200 AIMD trajectories of (left) $CH_4^+(\nu_3 = 1)$, (center) cold CH_4^+, and (right) $CH_4^+(\nu_4 = 1)$; Time where mean (dotted green curve) first crosses zero and reaches minimum are shown (dotted white lines). (Bottom row) corresponding time-resolved spectrum and their IWAE (solid black curve).

S8. SPECTRAL BROADENING EFFECT ON THE SIGNATURE IN XTAS SPECTRA

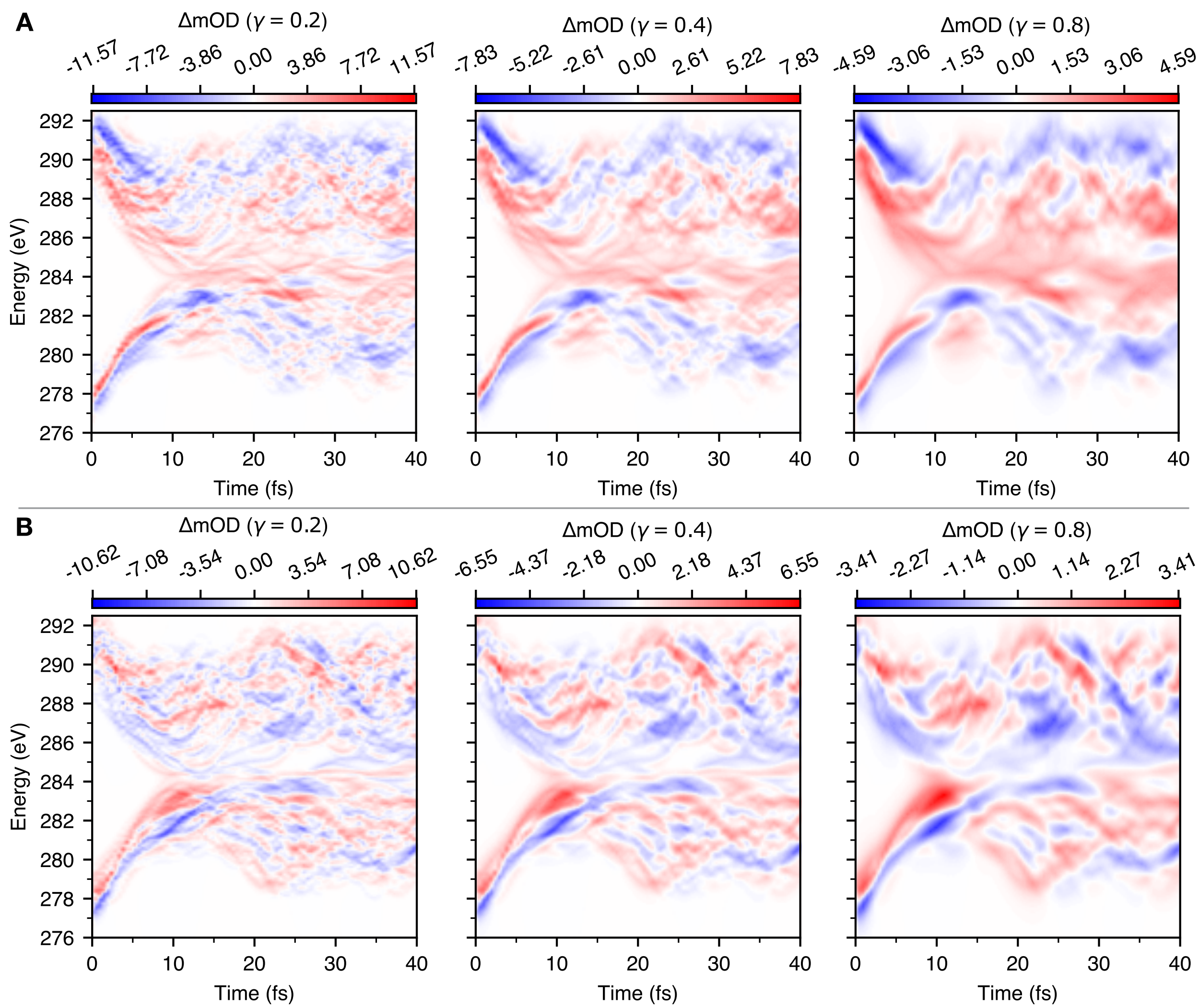

FIG. S6. Simulated difference X-ray transient absorption spectrum of CH_4^+ plotted using Lorentz broadening factors γ [0.2 eV (left), 0.4 eV (middle), and 0.8 eV (right)] following SFI of hot methane in its (A) $\nu_3 = 1$ and (B) $\nu_4 = 1$ vibrational states with respect to cold methane $\nu_i = 0$.

S9. SPECTRAL NOISE EFFECT ON THE SIGNATURE IN XTAS SPECTRA

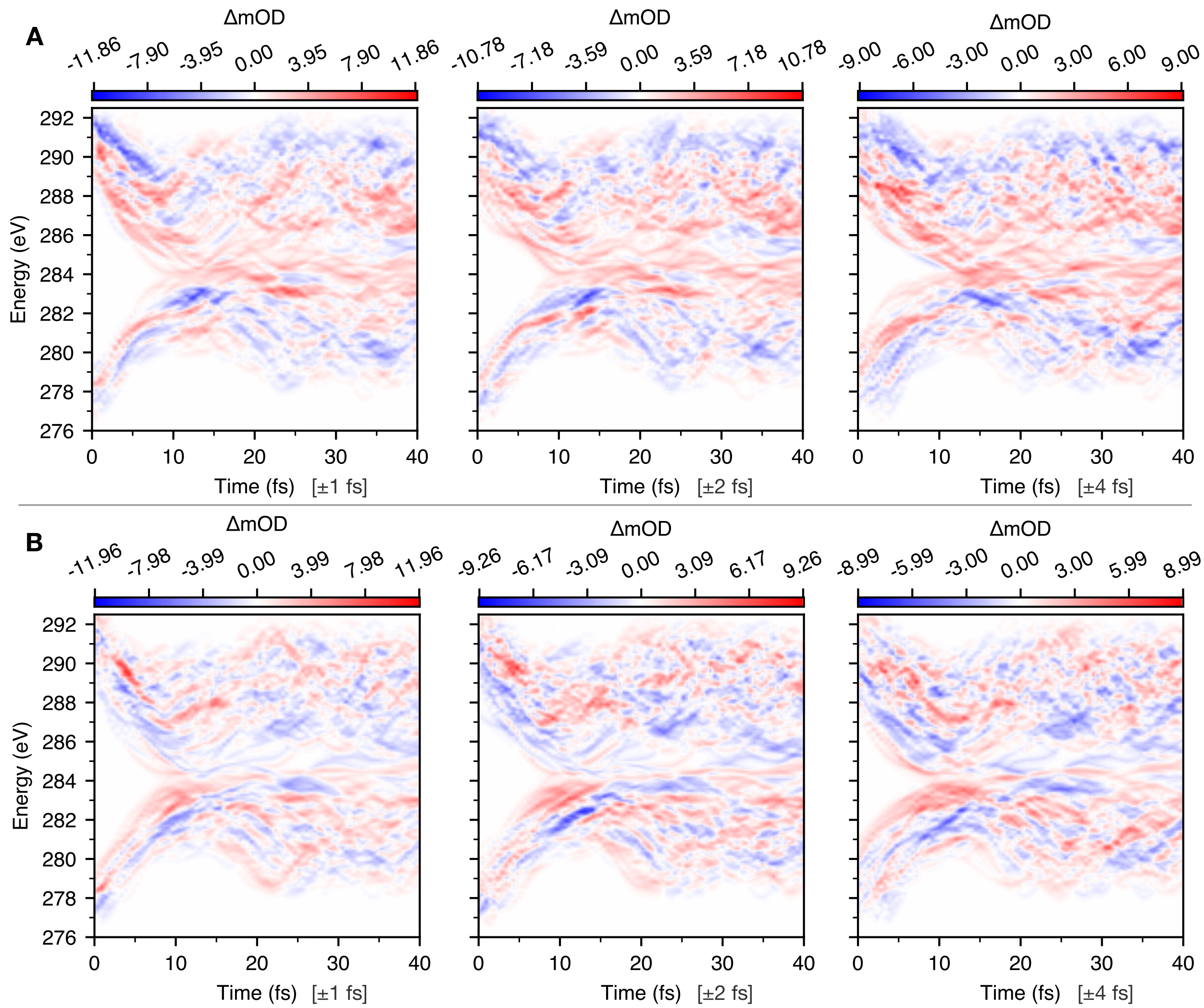

FIG. S7. Simulated difference X-ray transient absorption spectrum of CH_4^+ plotted by shifting trajectories' starting point randomly between a specific time-range [±1 fs (left), ±2 fs (middle), and ± 4 fs (right)] following SFI of hot methane in its (A) $\nu_3 = 1$ and (B) $\nu_4 = 1$ vibrational states with respect to cold methane $\nu_i = 0$.

REFERENCES

[1]Wigner, E. On the quantum correction for thermodynamic equilibrium. *Phys. Rev.* **1932**, *40*, 749–759.

[2]Heller, E. J. Time-dependent approach to semiclassical dynamics. *J. Chem. Phys.* **1975**, *62*, 1544–1555.

[3]Tannor, D. J.; Kosloff, R.; Rice, S. A. Coherent pulse sequence induced control of selectivity of reactions: Exact quantum mechanical calculations. *J. Chem. Phys.* **1986**, *85*, 5805–5820.

[4]Persico, M.; Granucci, G. An overview of nonadiabatic dynamics simulations methods, with focus on the direct approach versus the fitting of potential energy surfaces. *Theor. Chem. Acc.* **2014**, *133*, 1–28.

[5]Sun, L.; Hase, W. L. Comparisons of classical and Wigner sampling of transition state energy levels for quasiclassical trajectory chemical dynamics simulations. *J. Chem. Phys.* **2010**, *133*, 044313.

[6]Bunker, D. L.; Hase, W.-L. On non-RRKM unimolecular kinetics: Molecules in general, and CH3NC in particular. *J. Chem. Phys.* **1973**, *59*, 4621–4632.

[7]Hase, W. L.; Buckowski, D. G. Monte Carlo sampling of a microcanonical ensemble of classical harmonic oscillators. *Chem. Phys. Lett.* **1980**, *74*, 284–287.

[8]López, J. G.; McCoy, A. B. Classical treatments of quantum mechanical effects in collisions of weakly bound complexes. *Chem. Phys.* **2005**, *308*, 267–276.

[9]Malmqvist, P. Å.; Rendell, A.; Roos, B. O. The restricted active space self-consistent-field method, implemented with a split graph unitary group approach. *J. Phys. Chem.* **1990**, *94*, 5477–5482.

[10]Fleig, T.; Olsen, J.; Marian, C. M. The generalized active space concept for the relativistic treatment of electron correlation. I. Kramers-restricted two-component configuration interaction. *J. Chem. Phys.* **2001**, *114*, 4775–4790.

[11]Ma, D.; Li Manni, G.; Gagliardi, L. The generalized active space concept in multiconfigurational self-consistent field methods. *J. Chem. Phys.* **2011**, *135*.

[12]Vijayakumar, M.; Gopinathan, M. Study of the importance of relativistic, correlation, and relaxation effects on ionization energy of atoms by a relativistic and correlated local density method. *J. Chem. Phys.* **1995**, *103*, 6576–6580.

[13] Cooper, J. W. Near-threshold K-shell absorption cross section of argon: relaxation and correlation effects. *Phys. Rev. A* **1988**, *38*, 3417.

[14] Schmitt, A.; Schirmer, J. Molecular K-shell excitation spectra in the relaxed-core Hartree-Fock approximation. *Chem. Phys.* **1992**, *164*, 1–9.

[15] Nooijen, M.; Bartlett, R. J. Description of core-excitation spectra by the open-shell electron-attachment equation-of-motion coupled cluster method. *J. Chem. Phys.* **1995**, *102*, 6735–6756.

[16] Evangelista, F. A. A driven similarity renormalization group approach to quantum many-body problems. *J. Chem. Phys.* **2014**, *141*, 054109.

[17] Li, C.; Evangelista, F. A. Multireference theories of electron correlation based on the driven similarity renormalization group. *Annu. Rev. Phys. Chem.* **2019**, *70*, 245–273.

[18] Li, C.; Evangelista, F. A. Driven similarity renormalization group: Third-order multireference perturbation theory. *J. Chem. Phys.* **2017**, *146*, 124132.

[19] Evangelisti, S.; Daudey, J.; Malrieu, J. Qualitative intruder-state problems in effective Hamiltonian theory and their solution through intermediate Hamiltonians. *Phys. Rev. A* **1987**, *35*, 4930.

[20] Paldus, J.; Piecuch, P.; Pylypow, L.; Jeziorski, B. Application of Hilbert-space coupled-cluster theory to simple $(H_2)_2$ model systems: Planar models. *Phys. Rev. A* **1993**, *47*, 2738.

[21] Kowalski, K.; Piecuch, P. Complete set of solutions of multireference coupled-cluster equations: The state-universal formalism. *Phys. Rev. A* **2000**, *61*, 052506.

[22] Huang, M.; Evangelista, F. A. A study of core-excited states of organic molecules computed with the generalized active space driven similarity renormalization group. *J. Chem. Phys.* **2023**, *158*, 124112.

[23] Li, C.; Evangelista, F. A. Towards numerically robust multireference theories: The driven similarity renormalization group truncated to one-and two-body operators. *J. Chem. Phys.* **2016**, *144*, 164114.

[24] Evangelista, F. A. et al. Forte: A suite of advanced multireference quantum chemistry methods. *J. Chem. Phys.* **2024**, *161*, 062502.

[25] Dunning Jr, T. H. Gaussian basis sets for use in correlated molecular calculations. I. The atoms boron through neon and hydrogen. *J. Chem. Phys.* **1989**, *90*, 1007–1023.

[26] Woon, D. E.; Dunning Jr, T. H. Gaussian basis sets for use in correlated molecular calculations. V. Core-valence basis sets for boron through neon. *J. Chem. Phys.* **1995**, *103*, 4572–4585.

[27] De Jong, W. A.; Harrison, R. J.; Dixon, D. A. Parallel Douglas–Kroll energy and gradients in NWChem: Estimating scalar relativistic effects using Douglas–Kroll contracted basis sets. *J. Chem. Phys.* **2001**, *114*, 48–53.

[28] Verma, P.; Derricotte, W. D.; Evangelista, F. A. Predicting near edge X-ray absorption spectra with the spin-free exact-two-component Hamiltonian and orthogonality constrained density functional theory. *J. Chem. Theory Comput.* **2016**, *12*, 144–156.

[29] Kutzelnigg, W.; Liu, W. Quasirelativistic theory equivalent to fully relativistic theory. *J. Chem. Phys.* **2005**, *123*, 241102.

[30] Liu, W.; Peng, D. Exact two-component Hamiltonians revisited. *J. Chem. Phys.* **2009**, *131*, 031104.

[31] Smith, D. G. et al. PSI4 1.4: Open-source software for high-throughput quantum chemistry. *J. Chem. Phys.* **2020**, *152*, 184108.
